# Bathochromic Shift via C=O to C=S Substitution: A Far-Red Fluorogen for Multiplexed FLIM with FAST Fluorogen-Activating Protein

**DOI:** 10.3390/ijms27010023

**Published:** 2025-12-19

**Authors:** Aidar R. Gilvanov, Marina V. Molchanova, Svetlana A. Krasnova, Artur V. Eshtukov-Shcheglov, Andrey A. Mikhaylov, Sergey A. Goncharuk, Marina V. Goncharuk, Svetlana V. Sidorenko, Eugene G. Maksimov, Mikhail S. Baranov, Yulia A. Bogdanova

**Affiliations:** 1 Institute of Bioorganic Chemistry, Russian Academy of Sciences, Miklukho-Maklaya 16/10, Moscow 117997, Russiams.goncharuk@ibch.ru (S.A.G.);; 2 Laboratory of Medicinal Substances Chemistry, Institute of Translational Medicine, Pirogov Russian National Research Medical University, Ostrovitianova 1, Moscow 117513, Russia; 3 Department of Biology, Lomonosov Moscow State University, Leninskye Gory, Build. 12, Moscow 119234, Russia

**Keywords:** fluorogen-activating proteins, FAST, fluorogen, fluorescence, FLIM

## Abstract

The palette of the fluorogen-activating protein FAST expanded into the far-red region by the development of a novel fluorogen, **HBTR-3,5-DOM**. This was achieved through a C=O to C=S substitution in the classic hydroxybenzylidene-rhodanine core, which induced a bathochromic shift of over 100 nm. The complexes of **HBTR-3,5-DOM** with FAST variants pFAST and F62L are characterized by absorption and emission maxima at 640–650 nm and ~670 nm, respectively, and are found to exhibit distinct fluorescence lifetimes. The fluorogen is successfully applied in genetically encoded live-cell imaging together with these FAST variants for various subcellular structures. Furthermore, its potential for multiplexed imaging is demonstrated by the simultaneous discrimination of two targeted proteins using fluorescence lifetime imaging microscopy (FLIM).

## 1. Introduction

Fluorescence lifetime imaging microscopy (FLIM) is an emerging method for biological and medical studies [1]. FLIM exploits the fluorescence lifetime of fluorophores and enables detection of spectrally similar fluorescent tags using a single excitation source, making it an excellent platform for multiplexed imaging. Incorporation of additional spectral channels can further expand multiplexing capabilities. For biological applications, the far-red spectral region is particularly attractive due to minimal autofluorescence and reduced phototoxicity of excitation light [2].

Various synthetic probes, fluorescent proteins (FPs), and ligands for self-labeling proteins (SLPs) with far-red emission are available [3,4,5,6]. Synthetic probes offer simplicity and versatility; however, some exhibit non-specific labeling [7] and may require multiple staining protocols for multiplexed imaging [8]. Genetically encoded tags such as classical FPs with autocatalytic chromophores and SLPs (e.g., HaloTag) provide high labeling specificity but have large molecular weights (27 and 33 kDa, respectively), which can interfere with the function of fusion proteins of interest [9,10,11]. Smaller existing FPs (phiLOV, miniGFP) require cofactors like flavin for fluorescence [12,13], making them dependent on cofactor availability and limiting their color palette.

Fluorogen-activating proteins [14,15] present an attractive alternative. These chemogenetic tags lack intrinsic chromophores and rely on fluorogens—small molecules that are non-fluorescent in solution but become highly emissive upon binding to the protein. Fluorogen-activating and absorption-shifting tag (FAST) is a well-established fluorogen-activating protein with a molecular weight of only 14 kDa [16]. FAST binds its fluorogen ligands non-covalently, allowing fluorogen exchange between the binding pocket and surrounding medium, thereby enhancing tag photostability [17]. Numerous FAST variants and fluorogens have been developed for diverse microscopy applications [18,19,20,21,22,23]. Recent studies demonstrated that FAST-based systems enable multiplexed FLIM in the green-to-yellow [24,25] (525–575 nm) and red [26] (600–650 nm) spectral regions. Here, we characterize complexes of pFAST [20] and FAST-F62L [21] variants with the novel far-red arylidene-thiazolidine-2,4-dithione fluorogen **HBTR-3,5-DOM** (emission maximum at 670 nm) and evaluate its potential for multiplexed FLIM imaging.

## 2. Results and Discussion

**Figure 1:**
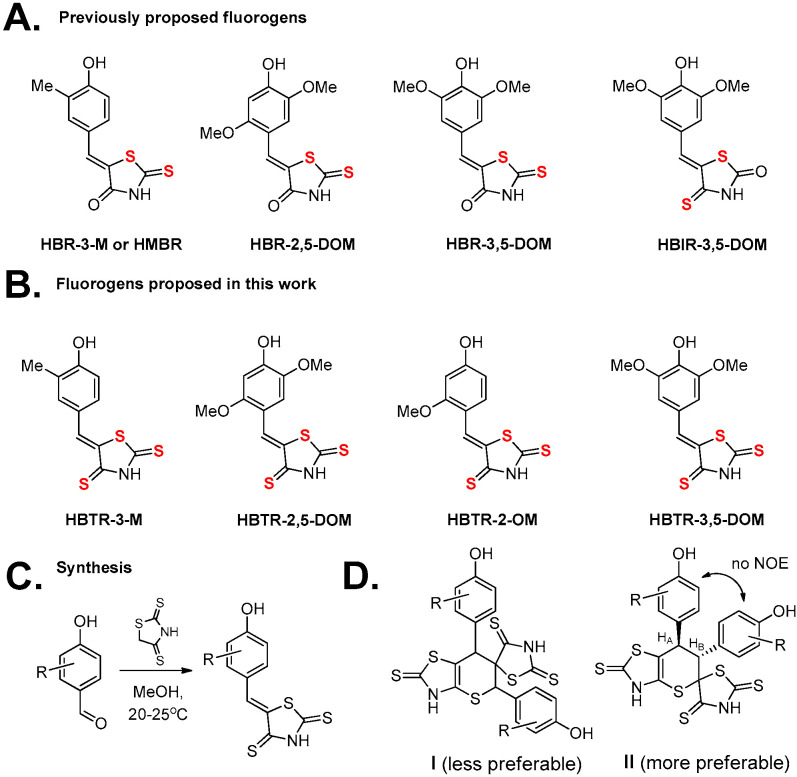
FAST fluorogens. (**A**) Structures of previously proposed fluorogens. (**B**) Structures of fluorogens proposed in this work. Sulphur atoms presented in fluorogens structures (**A**,**B**) are highlighted in red. (**C**) Synthesis of proposed fluorogens. (**D**) Two possible structures of product’s dimers.

A major focus of our work on developing new ligands for fluorogen-activating proteins is expanding their color palette. In our previous study on a lipocalin-based fluorogen-activating protein [27], we demonstrated that replacing the C=O group in the imidazolone moiety with a C=S group induces a significant bathochromic shift [28]. This shift is comparable to, or even exceeds, the effect achieved by introducing more complex conjugated fragments [27], while having a considerably smaller impact on the overall molecular structure and, consequently, the nature of protein binding. In the present study, we aimed to apply a similar modification to previously reported ligands of the fluorogen-activating protein FAST—hydroxybenzylidene rhodanines (**HBR** family, Figure 1) [16,17,18,19,20]. While a related modification has been previously explored, resulting in compounds containing one oxygen atom (iso-rhodanines, **HBIR** family) [20], we instead proposed the simultaneous introduction of three sulfur atoms into the heterocyclic fragment.

For this modification, we used four key fluorogens [16,17,18,19,20] of the FAST protein, each containing different substituents at the second, third, and fifth positions. This resulted in the creation of derivatives called **HBTR** (derived from Hydroxy-Benzylidene-Thio-Rhodanine). The structure and synthesis method are shown in Figure 1.

The target compounds were synthesized by condensing the corresponding 4-hydroxybenzaldehydes with commercially available thiazolidine-2,4-dithione (CAS 4303-27-9). This key reagent can also be readily prepared from the even more accessible rhodanine (2-thioxothiazolidin-4-one; detailed procedures are provided in the Section 3). Although the condensation yields ranged from 26% to 51%, all compounds were obtained in a pure, crystalline form directly from the reaction mixtures, eliminating the need for chromatographic purification.

The introduction of the sulfur atom led to unexpected behavior in the created substances. Thus, NMR analysis revealed their reversible dimerization at high concentration in solutions. Specifically, the ^1^H and ^13^C NMR spectra of compound **HBTR-3,5-DOM** at 33 mg/mL concentration exhibited two sets of signals in an approximate ratio of 1:0.4 (Figures S15.7 and S15.8). A double set of signals for the minor form indicates asymmetrical dimerization of the substance. The reversibility of this process was confirmed by diluting the concentrated sample ten-fold, which resulted in the ^1^H NMR spectrum showing a single set of signals corresponding exclusively to the initial monomeric form (Figure S15.9). Based on the literature data [29,30], the most plausible mechanism for this transformation is a [2 + 4] cycloaddition, which could proceed via two pathways to yield corresponding products **I** and **II** (Figure 1). This dimerization was observed for compounds **HBTR-3-M** and **HBTR-3,5-DOM**, but was absent for **HBTR-2-OM** and **HBTR-2,5-DOM**. This suggests that a substituent at the second (ortho to azolone moiety) position sterically hinders the process.

A detailed analysis of the NMR spectra (Figures S15.7–S15.16) allowed us to reliably determine the structure of the resulting dimer. On the one hand, the multiplet structure of H_A_ and H_B_ (Figure 1) signals at 4.96 ppm and 5.40 ppm (both are singlets) indicated the skeleton **I** rather than the skeleton **II**. The absence of correlations between protons H_A_ and H_B_ in the ^1^H,^1^H-COSY spectrum (Figure S15.13) also indicated **I** structure. However, in the ^1^H,^1^H-NOESY (Figure S15.14) spectrum clear COSY-type cross-peaks were observed between corresponding protons instead of NOE-correlation. It might indicate an extremely small value of the spin–spin coupling constant. In addition, in the ^1^H, ^13^C-HMBC (Figure S15.16) spectrum, a key correlation was observed between HB and C=S (Figure S15.11) while the cross-peak between HA and C=S was absent. Thus, combined analysis of the 1D and 2D NMR spectra allowed us to suggest a structure of the compound corresponding to **II** skeleton. Determination of the relative stereochemical configuration was based on the analysis of the ^1^H, ^1^H-NOESY spectrum. In the ^1^H, ^1^H-NOESY spectrum, there are no NOE-correlations between aromatic ring groups. Thus, based on the obtained data, it can be assumed that the aromatic substituents are in the trans-configuration (pseudo-axial position). Therefore, the protons of the thiopyran fragment H_A_ and H_B_ are in the pseudo-equatorial position. The value of the dihedral angle θ between the protons H_A_ and H_B_ tends to 90°, the value of the spin–spin coupling constant tends to 0 Hz according to the Karplus equation. The assignment results for both monomeric and dimeric structures are presented in the Section S1 in Supplementary Materials.

Since the revealed dimerization occurs only at high concentrations (>10 mM), it should not interfere with fluorogenic labeling in living systems, which employs concentrations three orders of magnitude lower. However, the uniqueness of this phenomenon, which has few analogs in the literature, makes it potentially valuable for synthetic organic chemistry, where it could enable novel synthetic strategies.

With these four fluorogens in hand, we investigated their interactions with the fluorogen-activating protein FAST and its “promiscuous” variant pFAST, which exhibits broad specificity for fluorogens, including the sulfur-containing analog **HBIR-3,5-DOM** [20]. Among them, the derivative **HBTR-3,5-DOM** demonstrated the most significant fluorescence enhancement and the largest bathochromic shift in its spectral maxima (Tables S4.1 and S4.2). We therefore selected **HBTR-3,5-DOM** for further characterization against pFAST and a panel of FAST mutants (D65K, F62L, P68K, R52A, R52K, R52Y; see Sections S2 and S3 in Supplementary Materials), chosen for their previously reported variation in fluorescence lifetimes [24,26]. Upon binding, all tested proteins induced a substantial increase in **HBTR-3,5-DOM** fluorescence (Table S4.3). Furthermore, most interactions were characterized by favorable dissociation constants, ranging from 0.1 to 0.5 µM (Table S5.1, Figures S5.1–S5.8), highlighting their potential for fluorescent live-cell imaging: this affinity range ensures efficient complex formation at micromolar fluorogen concentrations while preserving the reversible binding necessary for photobleaching resistance through fluorogen replenishment [23]. In this regard, the parent protein FAST was excluded from further consideration since it demonstrates a dissociation constant greater than 1.5 µM. It should be noted that one of the important aspects of fluorogen binding to the FAST protein is the formation of a hydrogen bond between W94 and the oxygen atom of the corresponding azolone ring. Replacing this oxygen with a sulfur atom could potentially disrupt this mechanism. However, the results obtained demonstrate that the formation of the hydrogen bond between the indole fragment of tryptophan and the sulfur atom is also possible. Thus, the use of azol-thiones as fluorogens of the FAST protein is clearly promising for the further search for new ligands.

To select optimal proteins for further multiplex FLIM experiments we subjected complexes of FAST variants with **HBTR-3,5-DOM** fluorogen to time-correlated single photon counting (TCSPC) spectroscopy. As a result, for most of the tested FAST variants, the lifetime value was about 2 ns, with the exception of pFAST and FAST-F62L variants whose lifetime value was about 2.2 and 1.5 ns, respectively (Table 1 and Table S6.1). In most cases, except for the complexes of FAST-P68K and FAST-R52Y variants, we observed acceptable χ^2^ values using monoexponential fitting, without further significant improvement with the biexponential model (Table S6.1, Figures S6.1–S6.14). Considering the difference in lifetime values we selected pFAST and FAST-F62L variants for further experiments. Notably, F62L has emerged as the preferred FAST variant across FLIM multiplexing system we previously proposed [24,26]. This Phe62 substitution is positioned directly opposite the benzylidene moiety and can participate in pi-stack interactions or act as a bulky stabilizing group, while Leu62 lacks this capacity. Depending on the fluorogen substituents, this effect is more or less pronounced and reaches a maximum in the presence of two bulky methoxy groups.

**Figure 2:**
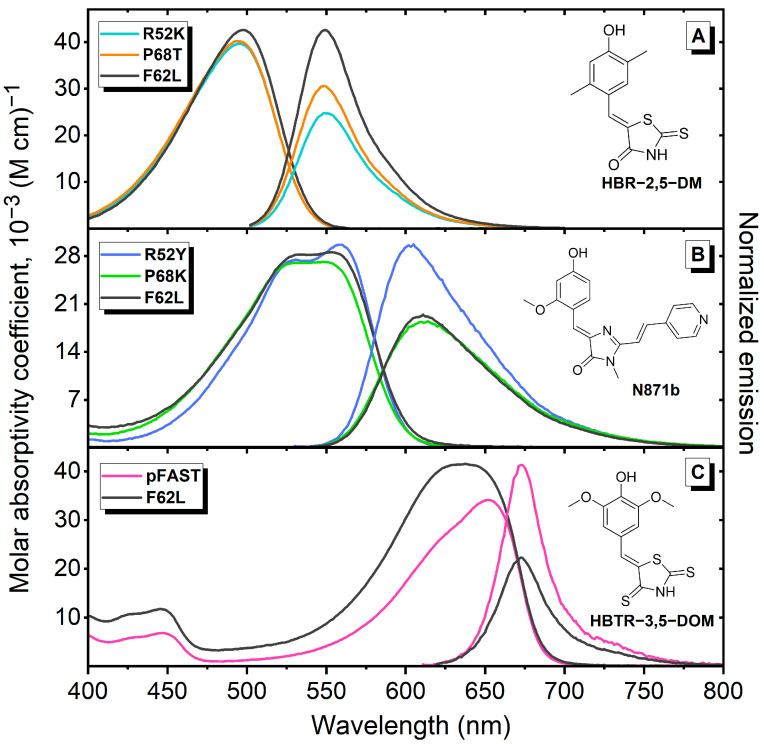
The absorption and emission spectra of various fluorogens in complexes with FAST variants used for FLIM imaging in [24,26] and the present article. (**A**) **HBR-2,5-DM** structure and spectra of its complexes with FAST-R52K, FAST-P68T, and FAST-F62L variants. (**B**) **N871b** structure and spectra of its complexes with FAST-R52Y, FAST-P68K, and FAST-F62L variants. (**C**) **HBTR-3,5-DOM** structure and spectra of its complexes with pFAST and FAST-F62L variants. The absorption spectra are normalized to the [protein–fluorogen] complex concentration and represented in the molar absorptivity coefficient scale. The emission spectra are normalized to the fluorescence quantum yield.

Next, we studied the spectral properties of **HBTR-3,5-DOM** complexes with pFAST and FAST-F62L in more detail. The introduction of a sulfur atom resulted in a significant bathochromic shift in the spectral maxima. The absorption maxima of the **HBTR-3,5-DOM** complexes with both proteins were located in the 640–650 nm region, with fluorescence maxima at approximately 670 nm. Thus, the sulfur atom incorporation induced a shift of more than 100 nm compared to the parent fluorogen **HBR-3,5-DOM** [31]. This spectral position makes **HBTR-3,5-DOM** one of the most red-shifted ligands known for the FAST protein. Previously, only **HPAR-3OM** and its analogs exhibited comparable absorption maxima [22,32]. Furthermore, fluorogens from that **HPAR** family contained an additional multiple bond in the arylidene fragment, which necessitated the development of specific FAST protein variants with an enlarged binding pocket [22,32]. In contrast, our proposed fluorogen is compatible with parent FAST protein and its point mutants. Despite the fact that the emission of fluorogens of the **HPAR** group turns out to be more long-wavelength due to the large Stokes shift, the **HBTR-3,5-DOM** we proposed is better suited for standard filters, such as Cy5. As observed for all previously reported FAST protein ligands, the spectral maxima of the complexes with the **HBTR-3,5-DOM** fluorogen matched those of its deprotonated form (Table 1 and Table S8.1, Figure 2, Figures S7.1, S7.2 and S8.1). This indicates that ligand binding promotes proton transfer from the phenolic hydroxyl group to Y42 and E46 residues, and that the introduction of a sulfur atom does not alter this fundamental mechanism. This behavior is a defining characteristic of the FAST protein (recall that the acronym stands for fluorescence-activating and absorption-shifting tag). The combination of fluorogen activation and this absorption shift significantly reduces off-target staining, as the unbound fluorogen not only exhibits weak fluorescence but also emits in a distinct spectral region.

We examined the pH dependence of the absorption spectra of **HBTR-3,5-DOM** and **HBTR-3-M** (Table S10.1, Figures S10.1–S10.4). Accurate pKa determination was hindered by poor solubility of the compounds (for this reason **HBTR-2-OM** and **HBTR-2,5-DOM** were excluded from this experiment entirely). The estimated pKa value of approximately 8 is in agreement with previously reported values for structurally similar compounds, e.g., **HBR-3,5-DOM** and **N871b** [33]. Thus, in the cellular environment, the free fluorogen exists predominantly in its protonated form. Interestingly, the molar absorption coefficient of the neutral form of compound **HBTR-3,5-DOM** is significantly lower than that of the deprotonated form (Table S8.1), potentially further reducing off-target labeling.

Additional experiments were performed in vitro to estimate time-resolved fluorescence anisotropy of **HBTR-3,5-DOM** dye in solution and in complexes with FAST. As expected (Figure S9.1), we observed rapid depolarization of free **HBTR-3,5-DOM** fluorescence in solution and dramatically slower rotation of the protein bound dye (~5 ns). High value of initial anisotropy of **HBTR-3,5-DOM** (0.4) indicates perfectly aligned absorption and emission dipoles.

The availability of spectral data, extinction coefficients, and fluorescence quantum yields (FQY) for different fluorogen–FAST pairs presents an opportunity to estimate fluorescence lifetimes using theoretical models. However, such calculations failed to predict the experimentally observed values. For instance, calculations performed using the Strickler–Berg equation [34] via an online calculator [35] gave values of 4.67 ns and 1.70 ns for the [pFAST–**HBTR-3,5-DOM**] and [FAST-F62L–**HBTR-3,5-DOM**] complexes, respectively. Both calculated lifetimes are significantly overestimated compared to the actual observed values. This discrepancy indicates the existence of additional non-radiative deactivation pathways for excited-state energy, which are not accounted by this model.

**Figure 3:**
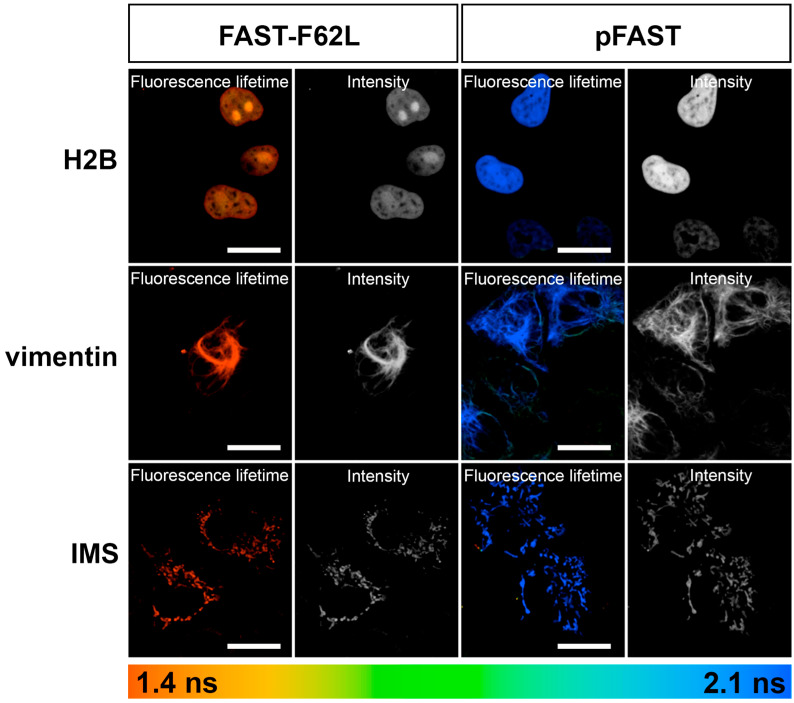
A. Live-cell FLIM images of **HBTR-3,5-DOM** fluorogen complexes with FAST-F62L and pFAST expressed as fusion constructs with histone H2B, intermediate filament protein vimentin, and intermembrane space localization signal (IMS). Color-coded fluorescence lifetime and total intensity images are represented for both variants. Scale bar—20 μm.

Next, we tested **HBTR-3,5-DOM** as a dye for fluorogen-activating tagging in live HeLa Kyoto cells using confocal fluorescence microscopy. HeLa Kyoto cells were transiently transfected with pFAST and FAST-F62L variants fused to subcellular localization signals (histone protein H2B, mitochondrial intermembrane space localization signal (IMS), and intermediate filament protein vimentin, Figure 3, total intensity images). **HBTR-3,5-DOM** efficiently penetrated several cell membranes, including plasma membrane, nuclear envelope, and outer mitochondrial membrane, specifically labeled the targeted compartments, and did not affect cell morphology. A fluorogen concentration of 1 μM proved to be suitable for staining. The cell toxicity test showed a low decrease in cell division rate even at a 10 times higher concentration (Table S11.1, Figure S11.1), demonstrating that the fluorogen can be used for prolonged live-cell FLIM experiments. Viability MTT-test showed no **HBTR-3,5-DOM**-related toxicity at staining concentration (Figure S11.2). It should be noted that the photostability of this tag is significantly lower than that of previously reported fluorogens (Figure S13.1); however, no phototoxicity-related changes in cell morphology were observed during imaging. Comparison of photobleaching curves for pFAST complexes with **HBTR-3,5-DOM** and **HBR-3,5-DOM** reveals that substitution of C=O with C=S in the azolone moiety adversely affects photostability.

Both variants exhibited monoexponential fluorescence decay in all cell compartments (Table S12.1, Figures S12.1–S12.7). The lifetime values obtained during in cellulo experiments were similar to in vitro measurements and clustered around 2.0 ns for pFAST and 1.4 ns for FAST-F62L (Table 2). Notably, although the difference in fluorescence lifetimes between H2B-fused complexes and those localized to vimentin and the mitochondrial intermembrane space (IMS) was modest, it was statistically significant (One-way ANOVA, *p* < 0.01, Tukey’s test, *p* < 0.01). A similar pattern was observed with the previously studied fluorogens **N871b** and **HBR-2,5-DM** [24,26]. The local microenvironment (pH, viscosity, or other factors) of each subcellular compartment likely influences the excited-state lifetime of the [protein–fluorogen] complex. Given the high degree of overlap between the excitation and emission spectra of the FAST variant complexes with **HBTR-3,5-DOM**, one might expect the possibility of homoFRET occurrence [36]; however, we did not observe any deviations in the fluorescence decay curves.

All FAST variants had distinct peaks on parameter histogram and distinct photon clusters on phasor plot (Figures S12.1–S12.7), which made them promising for FLIM multiplexing.

**Figure 4:**
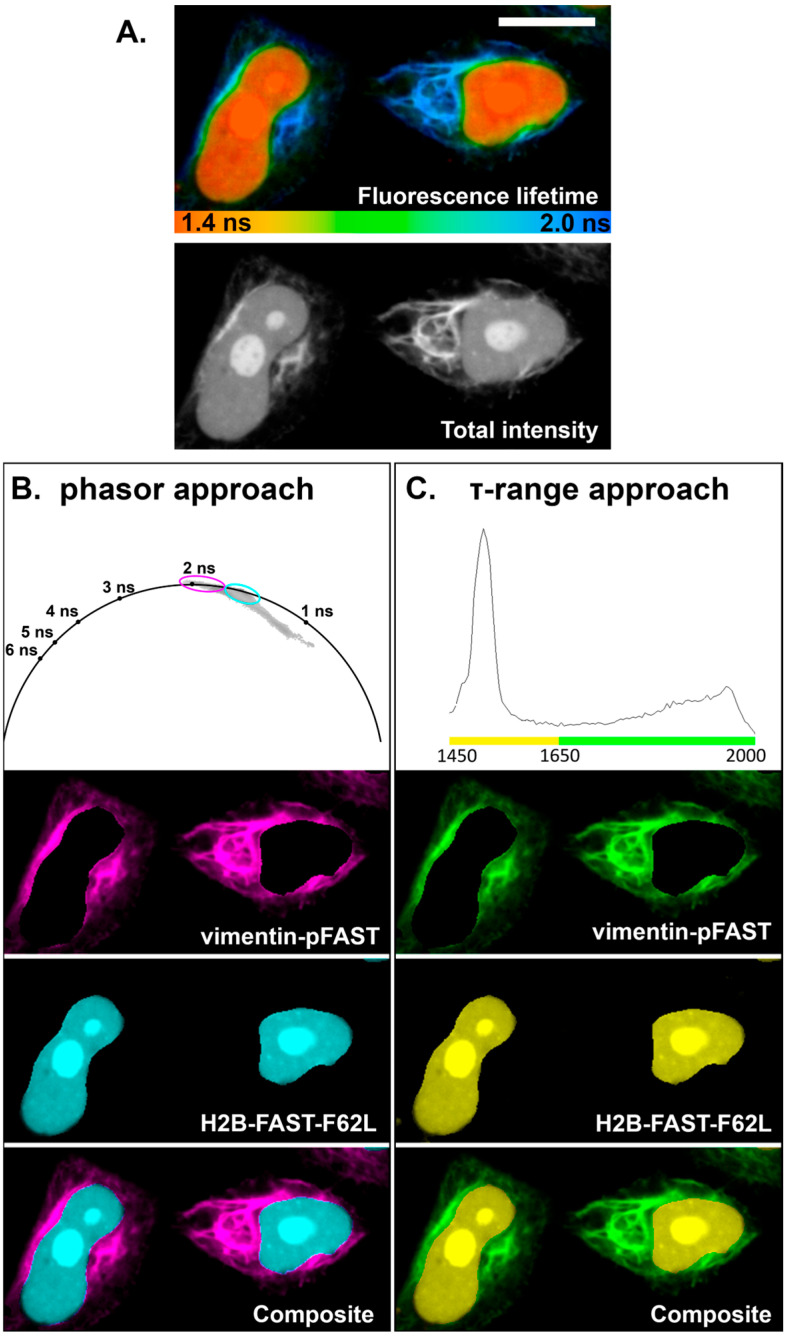
Dual-localization FLIM imaging of live HeLa Kyoto cells co-expressing vimentin-pFAST- and H2B-FAST-F62L fusion constructs in a complex with **HBTR-3,5-DOM** fluorogen. (**A**) Total intensity and color-coded FLIM image with monoexponential fit (τ) of **HBTR-3,5-DOM** in complex with FAST variants. Color coding for FLIM image is specified below the image. (**B**,**C**) Results of cellular compartments separation using phasor clusters (**B**) and τ ranges (**C**) specific to pFAST and F62L complexes with **HBTR-3,5-DOM**; the composite and individual localizations are shown. Cluster outline colors in the phasor plot correspond to the cellular compartment colors shown below. Scale bar—20 μm.

Finally, dual-localization FLIM-based discrimination was performed by co-expressing pFAST and FAST-F62L as fusion constructs targeting vimentin and H2B, respectively, in live HeLa Kyoto cells (Figure 4). We used τ-range and phasor approaches for analysis as described previously [24,26] due to the presence of well-resolved lifetime peaks in the distribution histogram and clearly separated phasor clusters. τ-ranges and phasor clusters were defined using histograms of the lifetimes and phasor plots obtained earlier for H2B-pFAST and H2B-FAST-F62L complexes with **HBTR-3,5-DOM**. Both approaches allowed us to distinguish cell areas corresponding to the nucleus and cytoskeleton. Similar data were obtained for reciprocal H2B-pFAST and vimentin-FAST-F62L pair (Figures S12.8 and S12.9).

We attempted to implement a bi-exponential model with fixed τ_1_ and τ_2_ at expected pFAST and FAST-F62L complexes with **HBTR-3,5-DOM** values during the fitting procedure. Previously [24], this method allowed us to separate overlapping areas of vimentin and nucleus for [FAST variants–**HBR-2,5-DM**] by estimation of τ_1_ and τ_2_ amplitudes contribution at each pixel of image, however in case of [FAST variants**–HBTR-3,5-DOM**] the attempt was unsuccessful, indicating that monoexponential fluorescence decay of the [FAST variant–fluorogen] is an insufficient condition.

## 3. Materials and Methods

### 3.1. General

Stock solution of all fluorogens were prepared in molecular biology-grade DMSO (Sigma-Aldrich, St. Louis, MO, USA) at 10 mM concentration and maintained at −20 °C protected from light for a maximum of 3 months. The corresponding data visualization and analysis were performed using Origin 2021 software suite (OriginLab Corporation, Northampton, MA, USA).

### 3.2. Synthesis

#### 3.2.1. Instrumentation and Methods

NMR spectra were recorded on Avance III 800, Bruker Fourier 300 and Bruker AVANCE AV 600 (Bruker, Billerica, MA, USA) at 303 °K. Chemical shifts are reported relative to residue peaks of DMSO d6 (2.50 ppm for ^1^H and 39.5 ppm for ^13^C).

High-resolution mass spectra (HRMS) were recorded on a Triple TOF 5600+ by AB Sciex instrument using electrospray ionization (ESI) (AB Sciex, Framingham, MA, USA). The measurements were performed in a positive ion mode with interface capillary voltage of 5.5 kV. Ion source gas flow was set at 30 arb; curtain gas flow was set at 25 arb. A syringe injection was used for solutions in a mixture of methanol and aqueous formic acid (0.1% vol) at a flow rate of 100 µL/min. Nitrogen was applied as a dry gas; interface temperature was set at 200 °C.

IUPAC compound names were generated using ChemDraw Software 16.0.

NMR spectra of all obtained compounds are presented in Section S15 in Supplementary Materials.

#### 3.2.2. Thiazolidine-2,4-dithione [37]

The substance was obtained by the described method [37]: a mixture of 2-thioxothiazolidin-4-one (6.64 g, 0.05 mol) and P_2_S_5_ (5 g, 0.01 mol) was refluxed for 15 min in 50 mL dry 1,4-dioxane. Then 1.18 g active charcoal and 2.2 g zinc dust were added and the mixture was refluxed for 2 min, after which it was filtered. The product was washed with diethyl ether (2 × 25 mL) and dried in vacuo. Yellow-green solid. Yield 100% (7.45 g).

#### 3.2.3. Preparation of (Z)-5-(4-Hydroxy-benzylidene)thiazolidine-2,4-dithiones

Thiazolidine-2,4-dithione (1 mmol) and corresponding 4-hydroxybenzaldehyde (1 mmol) were dissolved in 3 mL of MeOH. The resulting mixture was stirred for one hour (compounds **HBTR-2-OM**, **HBTR-3,5-DOM**, **HBTR-2,5-DOM**) or for 7 days (compound **HBTR-3-M**) at room temperature and filtered. The product was washed with hot MeOH and dried in air.

##### (Z)-5-(4-Hydroxy-2-methoxybenzylidene)thiazolidine-2,4-dithione (**HBTR-2-OM**)

Yield 47% (136 mg). Black solid. Melting point above 250 °C with decomposition. Only monomeric form observed in NMR spectra. ^1^H NMR (800 MHz, DMSO) δ 14.55 (s, 1H), 10.78 (s, 1H), 8.16 (s, 1H), 7.33 (d, J = 8.7 Hz, 1H), 6.57 (dd, J = 8.7, 2.3 Hz, 1H), 6.52 (d, J = 2.3 Hz, 1H), 3.87 (s, 3H). ^13^C NMR (201 MHz, DMSO) δ 196.7, 195.4, 163.8, 161.7, 131.7, 131.5, 129.5, 114.1, 109.5, 99.5, 55.9. HRMS (ESI) m/z [M+H]+ calcd for C_11_H_10_NO_2_S_3_: 283.9868, found: 283.9873.

##### (Z)-5-(4-Hydroxy-3-methylbenzylidene)thiazolidine-2,4-dithione (**HBTR-3-M**)

Yield 26% (70 mg). Violet solid. Melting point above 250 °C with decomposition. Both monomeric (major) and dimeric (minor) forms were observed in NMR spectra. A description of the spectra of the monomeric form is given below. ^1^H NMR (800 MHz, DMSO) δ 14.67 (s, 1H), 10.63 (s, 1H), 7.77 (s, 1H), 7.45 (d, J = 2.4 Hz, 1H), 7.42 (dd, J = 8.4, 2.4 Hz, 1H), 6.97 (d, J = 8.4 Hz, 1H), 2.18 (s, 3H). ^13^C NMR (75 MHz, DMSO) δ 196.8, 195.5, 159.8, 136.8, 134.5, 131.7, 130.0, 126.0, 124.6, 116.0, 15.8. HRMS (ESI) m/z [M+H]+ calcd for C_11_H_10_NOS_3_: 267.9919, found: 267.9923.

##### (Z)-5-(4-Hydroxy-2,5-dimethoxybenzylidene)thiazolidine-2,4-dithione (**HBTR-2,5-DOM**)

Yield 41% (129 mg). Black solid. Melting point above 250 °C with decomposition. Only monomeric form observed in NMR spectra. ^1^H NMR (800 MHz, DMSO) δ 14.57 (s, 1H), 10.66 (s, 1H), 8.18 (s, 1H), 6.90 (s, 1H), 6.63 (s, 1H), 3.84 (s, 3H), 3.82 (s, 3H). ^13^C NMR (75 MHz, DMSO) δ 196.3, 194.9, 156.7, 153.9, 142.7, 131.8, 129.3, 112.9, 111.8, 100.5, 56.2, 56.1. HRMS (ESI) *m*/*z* [M+H]+ calcd for C_12_H_12_NO_3_S_3_: 313.9974, found: 313.9977.

##### (Z)-5-(4-Hydroxy-3,5-dimethoxybenzylidene)thiazolidine-2,4-dithione (**HBTR-3,5-DOM**)

Yield 51% (160 mg). Black solid. Melting point above 250 °C with decomposition. HRMS (ESI) *m*/*z* [M+H]+ calcd for C_12_H_12_NO_3_S_3_: 313.9974, found: 313.9978. Both monomeric (major) and dimeric (minor) forms were observed in NMR spectra. Monomeric form: ^1^H NMR (600.13 MHz, DMSO-d6) δ ppm: 3.85 (s, 6H), 6.98 (s, 2H), 7.83 (s, 1H), 9.75 (br. s, 1H), 14.70 (br. s, 1H). ^13^C NMR (150.9 MHz, DMSO-d6) δ ppm: 56.1, 109.2, 123.9, 130.7, 137.0, 140.4, 148.4, 195.3, 196.5. Dimeric form: ^1^H NMR (600.13 MHz, DMSO-d6) δ ppm: 3.69 (s, 6H), 3.70 (s, 6H), 4.96 (s, 1H), 5.40 (s, 1H), 6.60 (s, 2H), 6.84 (s, 2H), 8.62 (br. s, 1H), 8.72 (br. s, 1H), 13.54 (br. s, 1H), 13.79 (br. s, 1H). ^13^C NMR (150.9 MHz, DMSO-d6) δ ppm: 54.1, 54.4, 56.1, 56.2, 82.4, 107.3, 108.9, 118.4, 122.3, 123.7, 127.7, 136.5, 136.8, 147.1, 147.5, 187.1, 201.6, 207.0.

### 3.3. DNA Cloning

The bacterial expression system based on pET24b(+) was utilized for recombinant protein production in *E. coli*. All plasmid constructs containing target genes cloned into pET24b(+) were obtained from Cloning Facility (Moscow, Russia). Each construct was designed with a C-terminal polyhistidine tag (GGGHHHHHH) for purification purposes. FAST variant sequences are listed in Table S2.1.

Coding sequences of pFAST and F62L FAST variants were obtained from Cloning Facility (Moscow, Russia) as Level 0 vectors compatible with the MoClo cloning system [38]. CMV promoter, SV40 poly(A) signal, and subcellular localization tags (H2B, mitochondrial intermembrane space localization signal (IMS), and vimentin), and Level 0 plasmids were taken from our in-house library. MoClo cloning assembly was carried out using Eco31I restriction enzyme (Thermo Scientific, Waltham, MA, USA) and T4 DNA Ligase (Evrogen, Moscow, Russia).

All DNA constructs were validated using Sanger sequencing.

### 3.4. Proteins Production and Purification

Expression and purification of the FAST variants (pFAST, D65K, F62L, P68K, R52A, R52K, R52Y) were performed as described previously [24,39]. A chemically competent BL21(DE3) *E. coli* strain was transformed with pET24b(+) plasmids encoding FAST variants and was cultivated until OD600 ~0.6 in M9 minimal salts medium at 37 °C and 250 rpm. Protein synthesis was induced using 0.25 mM of isopropyl β-d-1-thiogalactopyranoside (IPTG) followed by 4–5 h of post-induction cultivation. The bacterial cells were pelleted by centrifugation and lysed on ice by ultrasonication with Sonopuls HD GM2200 ultrasonic desintegrator (Bandelin electronic, Berlin, Germany) equipped with titanium tapered tip KE 76. The FAST variants carrying a C-terminal polyhistidine tag were purified using Ni^2+^ chelate affinity chromatography. The target proteins were dialyzed against 1× PBS buffer with 1 mM EDTA at 4 °C followed by concentration using 10 kDa MWCO Amicon Ultra device (Merck Millipore, Darmstadt, Germany) and by gel-filtration on Superdex 75 Tricorn 10/300 (GE Healthcare, Uppsala, Sweden) column.

### 3.5. Screening In Vitro

To characterize protein–fluorogen interactions, samples containing 10 µM protein with 1 µM fluorogen were analyzed in PBS buffer (pH 7.4, Amresco, Radnor, PA, USA). The fluorescence enhancement upon binding was calculated as the ratio of the fluorescence intensity of the fluorogen–protein mixture (10 µM protein with 1 µM fluorogen) to the fluorescence intensity of the free fluorogen (1 µM). The value was recorded at four different excitation wavelengths: 580, 600, 620, and 640 nm. The data were obtained in a single experiment. Fluorescence intensity values exceeding the instrument’s detection limit were recorded as the maximum detectable signal. Enhancement values should not be interpreted quantitatively.

### 3.6. Dissociation Constants Determination

Dissociation constants Kd values were determined by titrating 0.1 µM protein solutions with increasing fluorogen concentrations in PBS (pH 7.4, Amresco, Radnor, PA, USA) at 25 °C. Fluorescence intensity changes were monitored at 580 nm excitation wavelength using a Tecan Infinite 200 Pro M plate reader (Tecan, Männedorf, Switzerland). The dissociation constants Kd were calculated using the least squares fit.

### 3.7. Optical Properties

#### 3.7.1. Instrumentation

UV-Vis absorption measurements were performed on a Varian Cary 100 spectrophotometer (Agilent, Santa Clara, CA, USA), while fluorescence spectra were obtained using an Agilent Cary Eclipse spectrofluorometer (Agilent, Santa Clara, CA, USA).

#### 3.7.2. Fluorogen Optical Properties in Different Solvents

Optical properties of the fluorogen **HBTR-3,5-DOM** in its free form were investigated in five solvents including water with pH 8 (adjusted with NaOH) using 10 µM solutions for both absorption and emission spectra.

#### 3.7.3. Spectrophotometric Determination of the pKa

Spectrophotometric titration was performed using 2.7 μM **HBTR-3,5-DOM** or 5 μM **HBTR-3-M** in 10 mM NaH_2_PO_4_. The initial pH of 3–4 was adjusted with 0.1 M HCl. After recording the initial absorption spectrum, the solution was titrated with NaOH solutions of varying concentrations (0.1 M, 1 M, and 3 M), and absorption spectra were acquired after each addition.

#### 3.7.4. Spectra of Complexes

Absorption and emission spectra were acquired using 5 µM and 0.5 µM fluorogen, respectively, mixed with excess protein in PBS buffer (pH 7.4, Amresco) to ensure α ≥ 95% complex saturation. Protein concentration was calculated as follows:

(1) $$\left[{Pr}_{0}\right]=\frac{K_{d}\times \left(\alpha \times \left[{Chr}_{0}\right]\right)}{\left[{Chr}_{0}\right]-\left(\alpha \times \left[{Chr}_{0}\right]\right)}+\left(\alpha \times \left[{Chr}_{0}\right]\right)$$

where K_d_—dissociation constant, [Chr_0_]—fluorogen total concentration, α—saturation level of the protein–fluorogen complex.

#### 3.7.5. Fluorescence Quantum Yields Determination

Quantum yield measurements were performed according to the established method [40] with Oxazine 1 serving as the fluorescence standard. Absorption and emission were acquired using 5 µM and 0.5 µM fluorogen, respectively, with sufficient protein added to ensure α ≥ 95% complex saturation (Equation (1)). The quantum yield was calculated as follows:

(2) $${FQY}_{x}={FQY}_{st}\times \frac{F_{x}}{F_{st}}\times \frac{1-10^{{-A}_{st}}}{1-10^{{-A}_{x}}}\times \frac{n_{x}^{2}}{n_{st}^{2}}$$

where F—area under the emission peak, A—absorbance at the excitation wavelength, n—refractive index of the solvent, and the subscripts x and st indicate the fluorogen and standard, respectively.

#### 3.7.6. Fluorescence Anisotropy Measurement

The fluorescence decay curves with picosecond time resolutions were collected by a time-correlated single photon counting (TCSPC) custom-built set-up comprising a single photon counting module SPC-150 paired with a hybrid detector HMP-100-40, with the power supply controlled by DCC-100 (all made by Becker & Hickl, Berlin, Germany). The sample excitation was performed with vertically polarized light at 635 nm using a picosecond laser (InTop, St. Petersburg, Russia) with a 26 ps pulse duration driven at a repetition rate of up to 50 MHz. A long-pass filter with a 650 nm wavelength (FEL0550, Thorlabs, Newton, NJ, USA) was used to cut off the excitation signal. Detection was performed at 675 nm using monochromator ML-44 (Solar Laser Systems, Minks, Belarus). The polarization was adjusted by ultra-broadband wire-grid polarizers (WP25M-UB, Thorlabs, Newton, NJ, USA). All measurements were performed at 25C in a temperature-control cuvette holder Qpod 2e (Quantum Northwest, Liberty Lake, WA, USA).

### 3.8. Fluorescence Lifetime Measurement In Vitro

Fluorescence lifetime measurements were carried out using 0.5 μM fluorogen solution in PBS (pH 7.4). The FAST variants (pFAST, D65K, F62L, P68K, R52A, R52K, R52Y) were added at concentrations ensuring a protein–fluorogen complex formation of α ≥ 0.95. The total protein concentrations for complexes were determined using Equation (1).

The lifetime data acquisition was carried out in PMMA transparent optical cuvettes (Sarstedt, Nümbrecht, Germany) with TCSPC mini-Tau fluorescence spectrometer (Edinburgh Instruments, Livingston, UK) in a 50 ns window divided into 2048 time channels. For fluorescence excitation, the EPLED-590 picosecond laser (Edinburgh Instruments, Livingston, UK) with a central emission wavelength of 589.1 nm and repetition rate of 20 Mhz was used. The photons were counted in the spectral range of 650/25 nm. The data processing was carried out using Fluoracle 2.5.1 software (Edinburgh Instruments, Livingston, UK) with IRF-based deconvolution.

### 3.9. Cytotoxicity Test

HeLa Kyoto cells were cultured in DMEM (PanEco, Moscow, Russia), with 10% (*v*/*v*) of fetal bovine serum (Biowest, Nuaillé, France), 50 U/mL of penicillin, and 50 μg/mL of streptomycin (PanEco, Moscow, Russia), and transferred to DMEM medium without phenol red (Sigma Aldrich, USA) containing **HBTR-3,5-DOM** (added from 10 mM stock solution in DMSO) and supplemented with 25 mM HEPES (Sigma, St. Louis, MO, USA) and 10% fetal bovine serum (Biowest, Nuaillé, France). The cytotoxicity test was performed using multiposition time-lapse imaging with the BZ-9000 fluorescence microscope (Keyence Corporation, Osaka, Japan) equipped with INUF-KI4-F1 incubator unit (Tokai Hit, Fujinomiya, Japan) and ELWD 20×/0.45 S Plan Fluor objective (Nikon Corporation, Tokyo, Japan). Bright-field images were acquired at 37 °C every 20 min for 24 h. The number of cells in the first and last frames and the number of initiated cell divisions were quantified.

### 3.10. MTT Test

HeLa Kyoto cells were seeded at a density of 5 × 10^3^ cells per well in DMEM (PanEco, Moscow, Russia) supplemented with 10% (*v*/*v*) of fetal bovine serum (Biowest, Nuaillé, France), 50 U/mL of penicillin, and 50 μg/mL of streptomycin (PanEco, Moscow, Russia). Cells were incubated with 1 or 5 μM **HBTR-3,5-DOM** (added from 10 mM stock solution in DMSO) for 18 h, after which 3-(4,5-Dimethylthiazol-2-yl)-2,5-Diphenyltetrazolium Bromide (MTT) solution in PBS (5 mg/mL) was added to achieve the final concentration of 0.45 mg/mL. Cells were incubated for 2 h at 37 °C and 5% CO_2_. An equal volume of acidified with HCl isopropanol was added to each well and mixed thoroughly. Absorbance was measured at 570 nm using a Tecan Infinite 200 Pro M plate reader (Tecan, Männedorf, Switzerland).

### 3.11. Cell Culture and Transfection

HeLa Kyoto cells were cultured in DMEM (PanEco, Moscow, Russia) with 10% (*v*/*v*) of fetal bovine serum (Biowest, Nuaillé, France), 50 U/mL of penicillin, and 50 μg/mL of streptomycin (PanEco, Moscow, Russia) at 37 °C and 5% CO_2_ and seeded onto 35 mm glass-bottomed culture dishes (SPL Life Sciences, Gyeonggi-do, Republic of Korea) 24 h before transfection. Cells were transiently transfected with plasmids expressing FAST variants fused to localization signals using FuGENE 6 transfection reagent (Promega, Madison, WI, USA) at a 3:1 FuGENE:DNA ratio following the manufacturer’s instructions.

### 3.12. Photostability Study

Photobleaching analysis of **HBTR-3,5-DOM** in complex with pFAST expressed as an H2B fusion construct was conducted in live HeLa Kyoto cells. Transfection was performed using FuGENE HD reagent (Promega, Madison, WI, USA) at a 3:1 FuGENE:DNA ratio following the manufacturer’s instructions. Twenty-four hours post-transfection, cells were incubated for 5 min in Hank’s Balanced Salt Solution (PanEco, Moscow, Russia) containing 10 mM HEPES (Sigma, St. Louis, MO, USA) and supplemented with 5 μM **HBTR-3,5-DOM**, **N871b**, or **HBR-DOM** (added from 10 mM DMSO stock). Imaging was performed using a Leica TCS SP2 confocal system mounted on a Leica DM IRE inverted fluorescence microscope (Leica, Wetzlar, Germany) equipped with an HCX PL APO Lbd.BL 63× 1.40 oil immersion objective and a helium-neon laser (543 nm); fluorescence emission was collected at 555–700 nm using a GaAsP detector (Bedford, NH, USA). Photobleaching was conducted by continuous scanning of a 55 μm^2^ region at 0.3 fps with 1.25 μW/cm^2^ laser power at 400 Hz. Images were captured as 1024 × 1024 pixel 8-bit lei files and processed using Fiji software (version 1.54f).

### 3.13. Fluorescence Lifetime Imaging Microscopy

FLIM of live HeLa Kyoto cells was performed at room temperature in 2 mL of Hank’s Balanced Salt Solution (PanEco, Moscow, Russia) containing 10 mM HEPES (Sigma, St. Louis, MO, USA) and 1 µM of **HBTR-3,5-DOM**. The Eclipse Ti2 microscope (Nikon, Tokyo, Japan) with a Nikon 60× 1.4 oil immersion lens (Nikon, Tokyo, Japan), equipped with the DCS-120 scanning confocal module, SPC-150 module, and HMP-100-40C detector (Becker & Hickl, Berlin, Germany) was used for data acquisition. A 640 nm picosecond laser BDS-SM-LS-101 (Becker & Hickl, Berlin, Germany) with 30 ps duration pulses and repetition rate of 50 MHz was used for the fluorescence excitation. The photon collection time was set to 120 s, and the average input laser power of 5–8% of maximum (<200 μW/cm^2^) was used. The detection was performed using a combination of ET485LP and ET660LP long-pass filters (Chroma, Bellows Falls, VT, USA). The control of the system and data acquisition were carried out with SPCM data acquisition software SPC-150 v.9.87 (Becker & Hickl, Berlin, Germany).

FLIM data processing and visualization was carried out as described previously [24,26] using SPCImage software 8.9 (Becker & Hickl, Berlin, Germany) and Fiji 1.54k (imagej.net). Briefly, a decay matrix with monoexponential fitting was generated for the entire imaged region in each case. Fluorescence lifetime (τ) value ranges or phasor clusters were determined for each [FAST variant**–HBTR-3,5-DOM**] complex based on lifetime distribution histograms or phasor plots, respectively. For dual-localization FLIM of cells co-expressing vimentin and H2B-FAST variant fusion constructs, these τ ranges or phasor clusters were used to discriminate cellular compartments.

## 4. Conclusions

In summary, we have developed a novel family of far-red fluorogens for the FAST fluorogen-activating protein by introducing a sulfur-for-oxygen substitution in the heterocyclic core of classic hydroxybenzylidene rhodanine ligands. This modification resulted in a prominent bathochromic shift of over 100 nm, achieving fluorescence emission in the far-red region without the need to engineer an enlarged binding pocket of the protein. The **HBTR-3,5-DOM** complexes with selected FAST variants pFAST and F62L exhibited optimal binding affinity for stable labeling and fluorogen exchange, and maintained the characteristic absorption shift upon binding, a hallmark of the FAST system that minimizes off-target fluorescence. The key achievement of this work is the distinct fluorescence lifetimes exhibited by these complexes, which were reliably reproduced in live cells, enabling clear discrimination in FLIM. We successfully demonstrated the practical utility of this system by performing specific labeling of various subcellular compartments and achieving FLIM based separation. The **HBTR-3,5-DOM** probe represents a significant expansion of the FAST toolkit with perspectives for FLIM multiplexed imaging in the biologically favorable far-red spectral window.

## Figures and Tables

**Table 1:** The optical properties of the [FAST variant–fluorogen] complexes.

Fluorogen	FAST Variant	K_d_, µM ^a^	ε, M^−1^·cM^−1 b^	Abs, nm ^c^	FQY, %	Em, nm ^d^	τ, ns ^e^
**HBR-2,5-DM ^1^**	FAST-R52K	0.046 ± 0.004	37,500	495	25	550	1.41 ^f^
	FAST-P68T	0.090 ± 0.007	38,500	494	31	549	1.73 ^f^
	FAST-F62L	0.12 ± 0.02	40,500	498	43	549	2.32 ^f^
**N871b** ^2^	FAST-R52Y	0.24 ± 0.02	29,000	558	29	605	3.06 ^f^
	FAST-P68K	0.22 ± 0.01	26,500	548	18	610	2.13 ^f^
	FAST-F62L	0.55 ± 0.08	27,500	552	19	611	2.44 ^f^
**HBTR-3,5-DOM**	pFAST	0.09 ± 0.01	34,000	651	33	673	2.21
	FAST-F62L	0.37 ± 0.09	41,500	638	18	673	1.53

^a^—represented as mean ± SD; ^b^—extinction coefficient; ^c^—absorption maximum; ^d^—emission maximum; ^e^—fluorescence lifetimes; ^f^—bi-exponential fit, intensity-weighted average lifetime; ^1^—[24]; ^2^—[21,26].

**Table 2:** Fluorescence lifetimes of pFAST and FAST-F62L complexes with **HBTR-3,5-DOM** obtained in cellulo. τ is fluorescence lifetime. SD is standard deviation. *n* represents number of individual cells taken for analysis.

FAST Variant	H2B, τ ± SD, ns	Vimentin, τ ± SD, ns	IMS, τ ± SD, ns
pFAST	2.03 ± 0.05 *n* = 30	1.98 ± 0.07 *n* = 30	1.95 ± 0.07 *n* = 30
FAST-F62L	1.47 ± 0.04 *n* = 30	1.43 ± 0.06 *n* = 22	1.37 ± 0.07 *n* = 30

## Data Availability

The original contributions presented in this study are included in the article/Supplementary Materials. Further inquiries can be directed to the corresponding author.

## Supplementary Materials

The following supporting information can be downloaded at: https://www.mdpi.com/article/10.3390/ijms27010023/s1.

## Abbreviations

The following abbreviations are used in this manuscript:

FLIM	Fluorescence lifetime imaging microscopy
SLP	Self-labeling protein
FAST	Fluorescence-Activating and Absorption-Shifting Tag
TCSPC	Time-correlated single-photon counting
IPTG	β-d-1-thiogalactopyranoside
IMS	Mitochondrial intermembrane space localization signal
FP	Fluorescent proteins
DMSO	Dimethyl sulfoxide
PBS	Phosphate-buffered saline
HRMS	High-resolution mass spectra
DMEM	Dulbecco’s Modified Eagle Medium
EDTA	Ethylenediaminetetraacetic acid
ESI	Electrospray ionization
