# Supplementary material for: Bathochromic Shift via C=O to C=S Substitution: A Far-Red Fluorogen for Multiplexed FLIM with FAST Fluorogen-Activating Protein
Source: Int J Mol Sci. 2025 Dec 19;27(1):23. doi: 10.3390/ijms27010023 (PMC12786233; doi:10.3390/ijms27010023)
Supplement: Supplementary file 1 [file ijms-27-00023-s001.zip › ijms-4008442-supplementary.pdf]

Content

1. NMR data for dimeric and monomeric forms of compound HBTR-3,5-DOM 1

2. Amino acid sequences 4

3. Proteins production and purification 5

4 Screening in vitro 6

5. Determination of affinity constants 9

6. Fluorescence lifetime screening in vitro 14

7. Optical properties of the [FAST variant-HBTR-3,5-DOM] complexes 22

8. Optical properties of the fluorogen HBTR-3,5-DOM in a free form 23

9. Fluorescence anisotropy 24

10. Spectrophotometric titration of fluorogens HBTR-3,5-DOM and HBTR-3-M 25

11. Cytotoxicity test 28

12. FLIM 31

13. HBTR-3,5-DOM photostability 41

14. References 42

15. Copies of NMR spectra 43

1. NMR data for dimeric and monomeric forms of compound HBTR-3,5-DOM

^{1}H NMR spectroscopic data in tables S1.1 and S1.2 below are reported as follows: chemical shift in ppm (H-position in chemical structure, multiplicity, spin-spin coupling constants J (Hz), integration intensity). The multiplicities are abbreviated with s (singlet), d (doublet), t (triplet), broad (br.), combinations thereof, and m (multiplet). In case of combined multiplicities, the multiplicity with the larger coupling constant is stated first. Except for complex and overlapping multiplets, where a resonance range is given, the chemical shift of all other symmetric signals is reported as the center of the resonance multiplet. ^{13}C NMR spectroscopic data in tables S1.1 and S1.2 below are reported as follows: chemical shift in ppm (C-position in chemical structure).

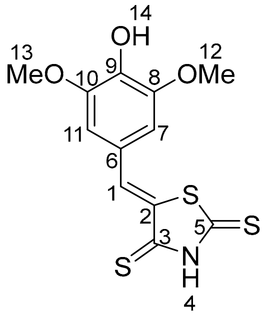

Figure S1.1. Atoms numeration of monomeric form of ***HBTR-3,5-DOM***.

Table S1.1. ^{1}H and ^{13}C NMR data of monomeric form of ***HBTR-3,5-DOM***.

**Position**	**^{13}C**		**^{1}H**
C1-H	137.0	7.83	s, 1H
C2	123.9	-	-
C3	195.3	-	-
N4-H	-	14.70	exchange with H_2O, 1H
C5	196.5	-	-
C6	130.7	-	-
C7-H<br>C11-H	109.2	6.98	s, 2H
C8<br>C10	148.4	-	-
C9	140.4	-	-
C12-H<br>C13-H	56.1	3.85	s, 6H
O14-H	-	9.75	exchange with H_2O, 1H

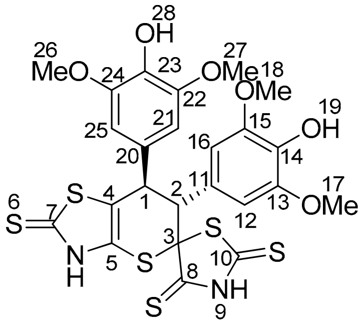

Figure S1.2. Atoms numeration of dimeric form of ***HBTR-3,5-DOM***.

Table S1.2. ^{1}H and ^{13}C NMR data of monomeric form of ***HBTR-3,5-DOM***.

**Position**	**^{13}C**		**^{1}H**
C1-H	54.4	5.40	s, 1H
C2-H	54.1	4.96	s, 1H
C3	82.4	-	-
C4*	118.4	-	-
C5*	127.7	-	-
S6-H*	-	13.54/13.79	exchange with H_2O, 1H
C7	187.1	-	-
C8	207.0	-	-
N9-H*	-	13.54/13.79	exchange with H_2O, 1H
C10	201.6	-	-
C11	123.7	-	-
C12*-H<br>C16*-H	108.9	6.60	s, 2H
C13<br>C15	147.1	-	-
C14	136.5	-	-
C17-H<br>C18-H	56.2	3.69	s, 6H
O19-H*	-	8.62/8.72	exchange with H_2O, 1H
C20	122.3	-	-
C21-H<br>C25-H	107.3	6.84	s, 2H

C22<br>C24	147.5	-	-
C23	136.8	-	-
C26-H<br>C27-H	56.1	3.70	s, 6H
O28-H*	-	8.62/8.72	exchange with H_2O, 1H

* - *signals can't be unambiguously assigned*

1. Amino acid sequences

Table S2.1. Amino acid sequences of FAST variants used in present work.

FAST (original)	(M)EHVAFGSEDIENTLAKMDDGQLDGLAFGAIQLDGDGNILQYNAAEGDITG<br>RDPKQVIGKNFFKDVAPGTDSPEFYGKFKEGVASGNLNTMFEWMIPTSRGPTK<br>VKVHM KKALSGDSYWVFVKRV(GGGHHHHHH)
pFAST[1]	(M)EHVAFGSEDIENTLA**N**MDD**E**QLD**R**LAFG**V**IQLDGDGNIL**L**YNAAEGDITG<br>RDPKQVIGKNFFKDVAPGTD**T**PEFYGKFKEG**A**ASGNLNTMFEW**T**IPTSRGPTK<br>VKVH**L**KKALSGD**R**YWVFVKRV(GGGHHHHHH)
FAST-D65K[2]	(M)EHVAFGSEDIENTLAKMDDGQLDGLAFGAIQLDGDGNILQYNAAEGDITG<br>RDPKQVIGKNFFK**K**VAPGTDSPEFYGKFKEGVASGNLNTMFEWMIPTSRGPTK<br>VKVHM KKALSGDSYWVFVKRV(GGGHHHHHH)
FAST-F62L[2]	(M)EHVAFGSEDIENTLAKMDDGQLDGLAFGAIQLDGDGNILQYNAAEGDITG<br>RDPKQVIGKN**L**FKDVAPGTDSPEFYGKFKEGVASGNLNTMFEWMIPTSRGPTK<br>VKVHM KKALSGDSYWVFVKRV(GGGHHHHHH)
FAST-P68K[2]	(M)EHVAFGSEDIENTLAKMDDGQLDGLAFGAIQLDGDGNILQYNAAEGDITG<br>RDPKQVIGKNFFKDVA**K**GTDSPEFYGKFKEGVASGNLNTMFEWMIPTSRGPTK<br>VKVHM KKALSGDSYWVFVKRV(GGGHHHHHH)
FAST-R52A[2]	(M)EHVAFGSEDIENTLAKMDDGQLDGLAFGAIQLDGDGNILQYNAAEGDITG<br>**A**DPKQVIGKNFFKDVAPGTDSPEFYGKFKEGVASGNLNTMFEWMIPTSRGPTK<br>VKVHM KKALSGDSYWVFVKRV(GGGHHHHHH)
FAST-R52K[2]	(M)EHVAFGSEDIENTLAKMDDGQLDGLAFGAIQLDGDGNILQYNAAEGDITG<br>**K**DPKQVIGKNFFKDVAPGTDSPEFYGKFKEGVASGNLNTMFEWMIPTSRGPTK<br>VKVHM KKALSGDSYWVFVKRV(GGGHHHHHH)
FAST-R52Y[2]	(M)EHVAFGSEDIENTLAKMDDGQLDGLAFGAIQLDGDGNILQYNAAEGDITG<br>**Y**DPKQVIGKNFFKDVAPGTDSPEFYGKFKEGVASGNLNTMFEWMIPTSRGPTK<br>VKVHM KKALSGDSYWVFVKRV(GGGHHHHHH)

1 - pFAST variant was described in work by Benaissa et al.[1]

2 - D65K, F62L, P68K, R52A, R52K, R52Y variants were described in work by Goncharuk et al.[2]

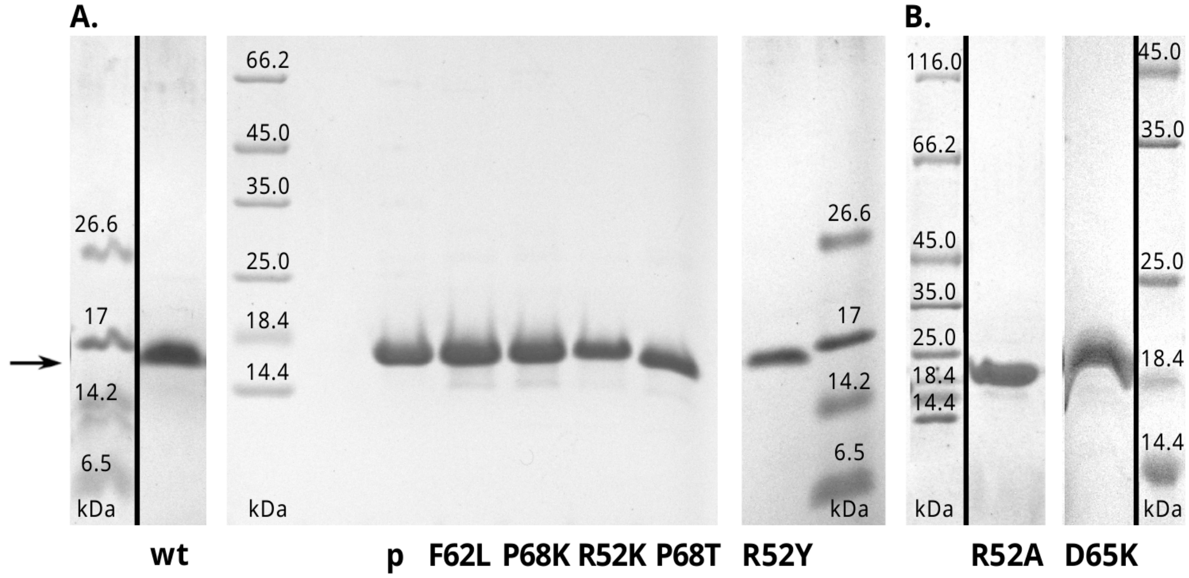

2. Proteins production and purification

Figure S3.1. Purified protein samples of FAST variants used in the work. Samples are loaded onto Tris-Tricine (**A**) or Tris-Glycine (**B**) SDS-page. wt – wild type of FAST (14.7 kDa), p – pFAST. Bands corresponding to the target protein are indicated by an arrow. Protein molecular weight markers are the following: 116.0, 66.2, 45.0, 35.0, 25.0, 18.4, 14.4 kDa, and 26.6, 17.0, 14.2, 6.5 kDa.

4. *Screening in vitro*

Table S4.1. Fluorescence enhancement of FAST with novel fluorogens.

**Compound**	**Structure**	**Enhancement***			
		**580 nm**	**600 nm**	**620 nm**	**640 nm**
**HBTR-2-OM**	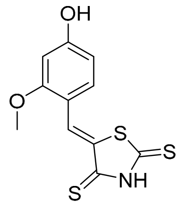	6.4	3.2	1.2	1.0
**HBTR-3,5-DOM**	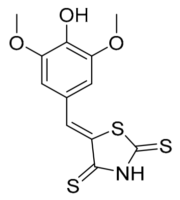	28.6	89.3	79.6	496.6
**HBTR-3-M**	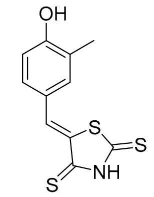	10.6	2.2	1.1	1.1
**HBTR-2,5-DOM**	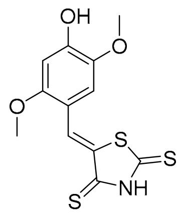	50.0	27.0	6.5	13.3

*The fluorescence enhancement upon binding was calculated as the ratio of the fluorescence intensity of the fluorogen-protein mixture (10 μM protein with 1 μM fluorogen) to the fluorescence intensity of the free fluorogen (1 μM). The value was recorded at four different excitation wavelengths: 580, 600, 620, and 640 nm. The data were obtained in a single experiment. Fluorescence intensity values exceeding the instrument's detection limit were recorded as the maximum detectable signal. Enhancement values should not be interpreted quantitatively.

Table S4.2. Fluorescence enhancement of pFAST with novel fluorogens.

Compound	Structure	Enhancement*			
		580 nm	600 nm	620 nm	640 nm
**HBTR-2-OM**	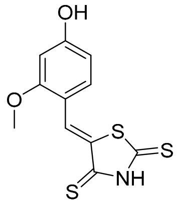	11.1	2.8	1.1	0.8
**HBTR-3,5-DOM**	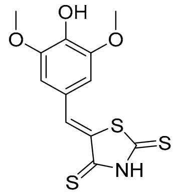	44.1	138.4	123.3	767.4
**HBTR-3-M**	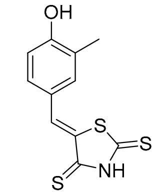	8.6	1.9	1.1	1.0
**HBTR-2,5-DOM**	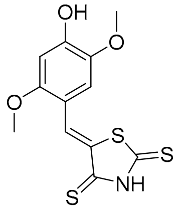	62.6	29.2	8.1	17.5

*The fluorescence enhancement upon binding was calculated as the ratio of the fluorescence intensity of the fluorogen-protein mixture (10 µM protein with 1 µM fluorogen) to the fluorescence intensity of the free fluorogen (1 µM). The value was recorded at four different excitation wavelengths: 580, 600, 620, and 640 nm. The data were obtained in a single experiment. Fluorescence intensity values exceeding the instrument's detection limit were recorded as the maximum detectable signal. Enhancement values should not be interpreted quantitatively.

Table S4.3. Fluorescence enhancement of FAST variants with **HBTR-3,5-DOM.**

**Compound**	**Protein**	**Enhancement***			
		**580 nm**	**600 nm**	**620 nm**	**640 nm**
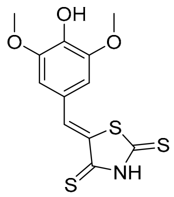 **HBTR-3,5-DOM**	FAST	28.6	89.3	79.6	496.6
	pFAST	44.1	138.4	123.3	767.4
	FAST-P68K	26.4	83.8	77.3	507.2
	FAST-D65K	34.6	108.0	94.2	563.6
	FAST-F62L	28.0	82.5	65.8	351.1
	FAST-R52Y	14.3	47.0	45.0	299.4
	FAST-R52A	27.0	94.3	97.0	732.4
	FAST-R52K	21.5	73.0	72.8	535.3

*The fluorescence enhancement upon binding was calculated as the ratio of the fluorescence intensity of the fluorogen-protein mixture (10 μM protein with 1 μM fluorogen) to the fluorescence intensity of the free fluorogen (1 μM). The value was recorded at four different excitation wavelengths: 580, 600, 620, and 640 nm. The data were obtained in a single experiment. Fluorescence intensity values exceeding the instrument's detection limit were recorded as the maximum detectable signal. Enhancement values should not be interpreted quantitatively.

5. Determination of affinity constants

Table S5.1. The dissociation constants values of the [FAST variant- **HBTR-3,5-DOM**] complexes.

**Compound**	**Structure**	**Protein**	**K_d, µM***
**HBTR-3,5-DOM**	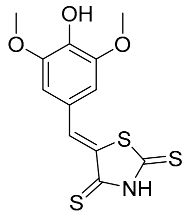	FAST	1.64 ± 0.43
		pFAST	0.09 ± 0.01*
		FAST-P68K	0.21 ± 0.07
		FAST-D65K	0.25 ± 0.06
		FAST-F62L	0.37 ± 0.09
		FAST-R52Y	0.50 ± 0.12
		FAST-R52A	0.23 ± 0.03
		FAST-R52K	0.44 ± 0.17

*All measurements were performed in a single measurment except for pFAST, for which three repetitions were performed

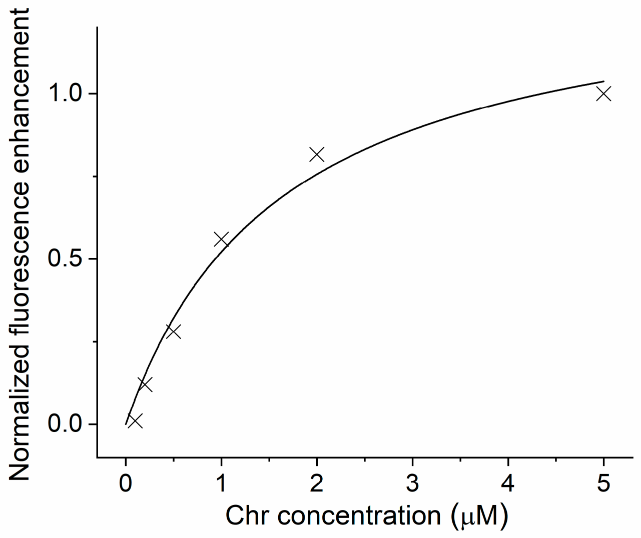

Figure S5.1. The titration curve observed for **HBTR-3,5-DOM** complex with FAST.

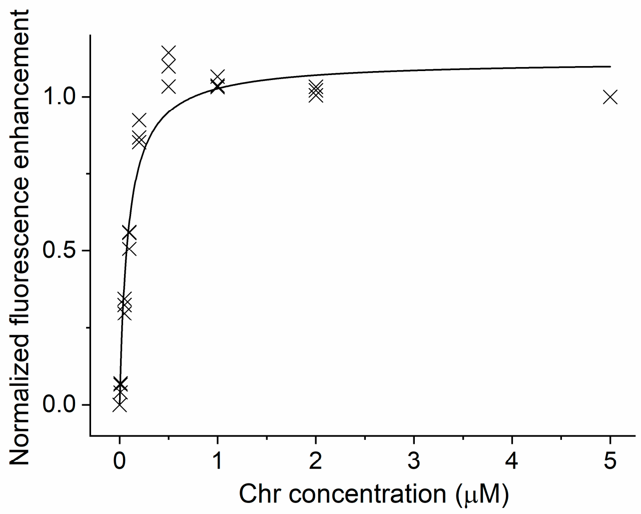

Figure S5.2. The titration curve observed for **HBTR-3,5-DOM** complex with pFAST.

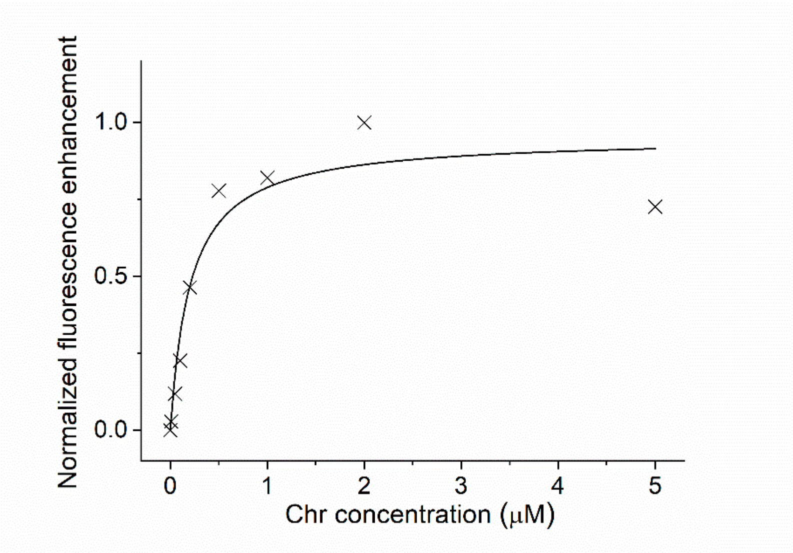

Figure S5.3. The titration curve observed for **HBTR-3,5-DOM** complex with FAST-P68K.

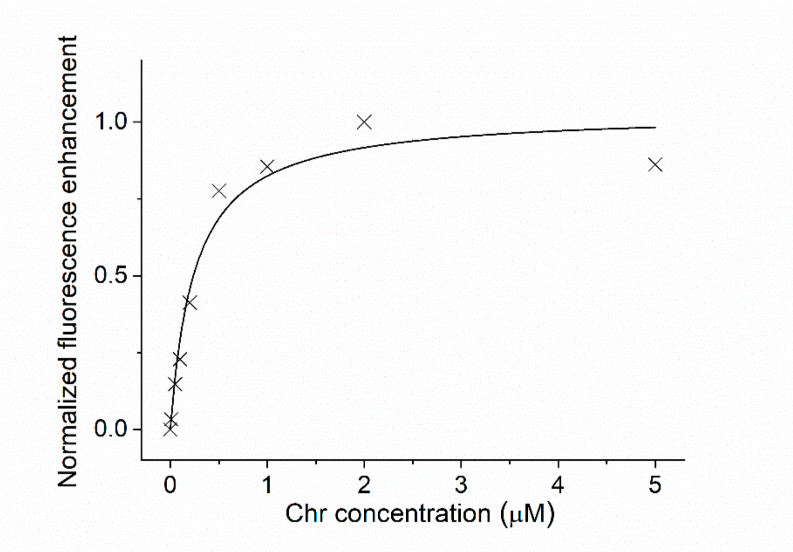

Figure S5.4. The titration curve observed for **HBTR-3,5-DOM** complex with FAST-D65K.

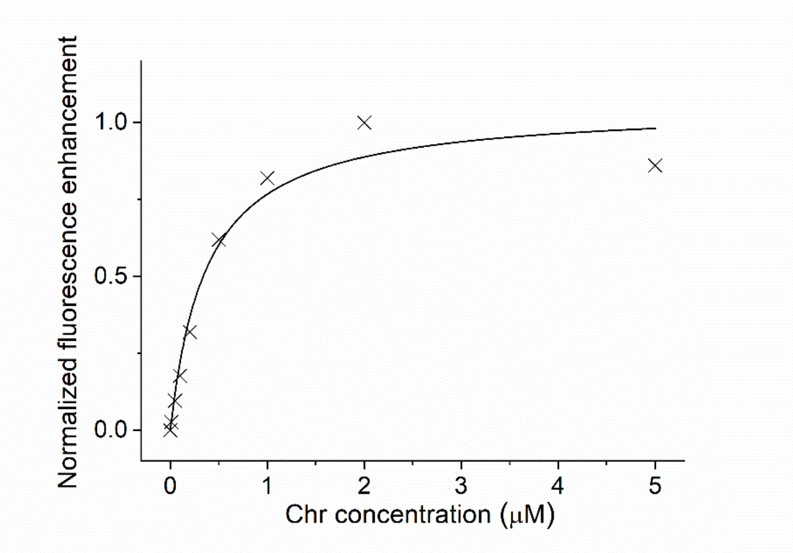

Figure S5.5. The titration curve observed for **HBTR-3,5-DOM** complex with FAST-F62L.

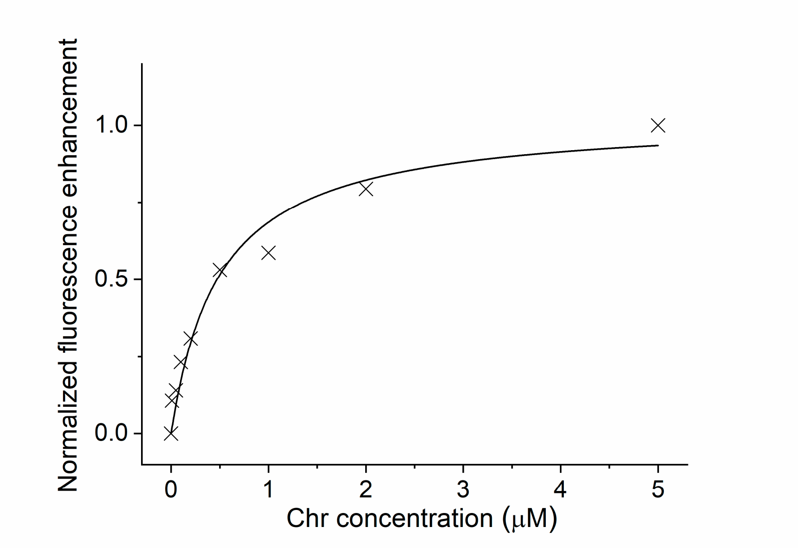

Figure S5.6. The titration curve observed for **HBTR-3,5-DOM** complex with FAST-R52Y.

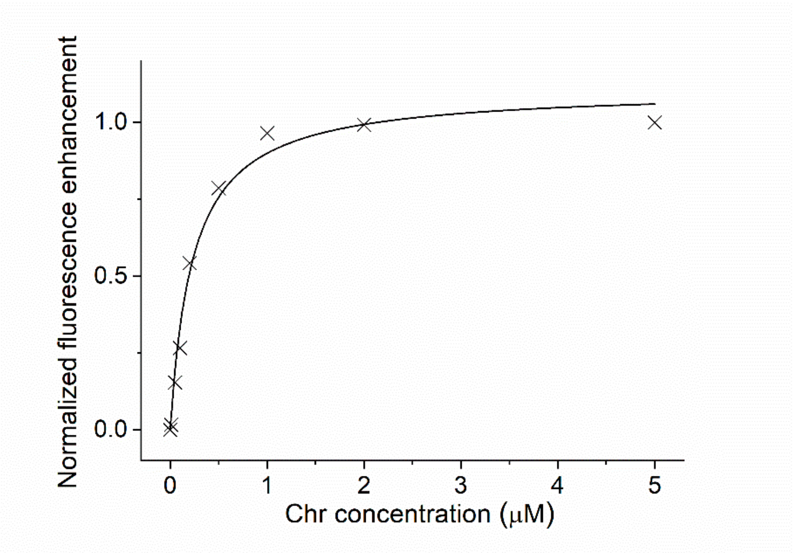

Figure S5.7. The titration curve observed for **HBTR-3,5-DOM** complex with FAST-R52A.

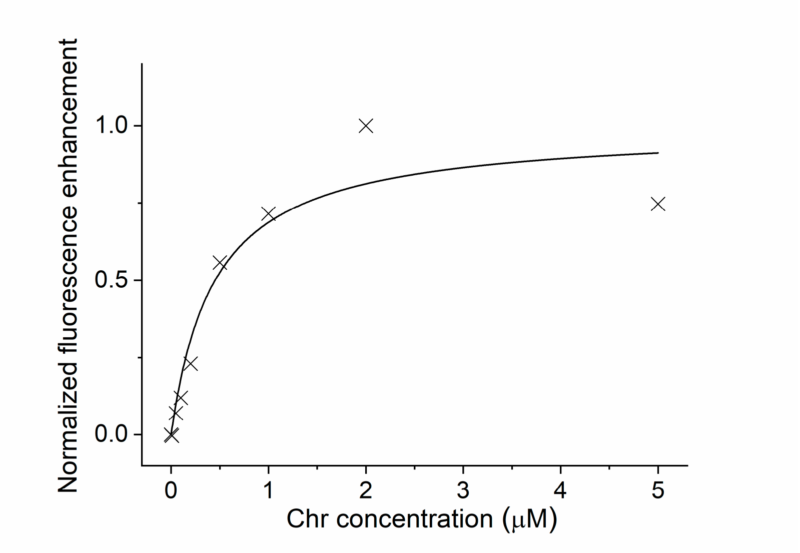

Figure S5.8. The titration curve observed for **HBTR-3,5-DOM** complex with FAST-R52K.

6. Fluorescence lifetime screening in vitro

Table S6.1. Comparison of mono-exponential and bi-exponential fit for lifetime data of [FAST variant-**HBTR-3,5-DOM**] complexes obtained *in vitro* with purified protein. τ_m is amplitude weighted average lifetime.

Fit model	FAST variant	τ_1, ns	A1, %	τ_2, ns	A2, %	τ_m, ns	χ^2
**Mono-exponential fit**	pFAST	2.210±0.004	-	-	-	-	1.172
	FAST-D65K	1.941±0.003	-	-	-	-	1.331
	FAST-F62L	1.533±0.003	-	-	-	-	1.302
	FAST-P68K	1.892±0.003	-	-	-	-	1.422
	FAST-R52A	2.093±0.004	-	-	-	-	1.264
	FAST-R52K	1.983±0.003	-	-	-	-	1.288
	FAST-R52Y	1.768±0.003	-	-	-	-	1.773
**Bi-exponential fit**	pFAST	0.158±0.076	4	2.231±0.005	96	2.148	1.128
	FAST-D65K	0.666±0.096	7	2.002±0.010	93	1.908	1.249
	FAST-F62L	0.564±0.080	10	1.597±0.010	90	1.494	1.211
	FAST-P68K	0.664±0.072	10	1.979±0.010	90	1.848	1.265
	FAST-R52A	0.473±0.080	5	2.141±0.007	95	2.058	1.178
	FAST-	0.759±0.083	9	2.072±0.011	91	1.954	1.149

	R52K						
	FAST-R52Y	0.703±0.036	21	1.971±0.012	79	1.705	1.182

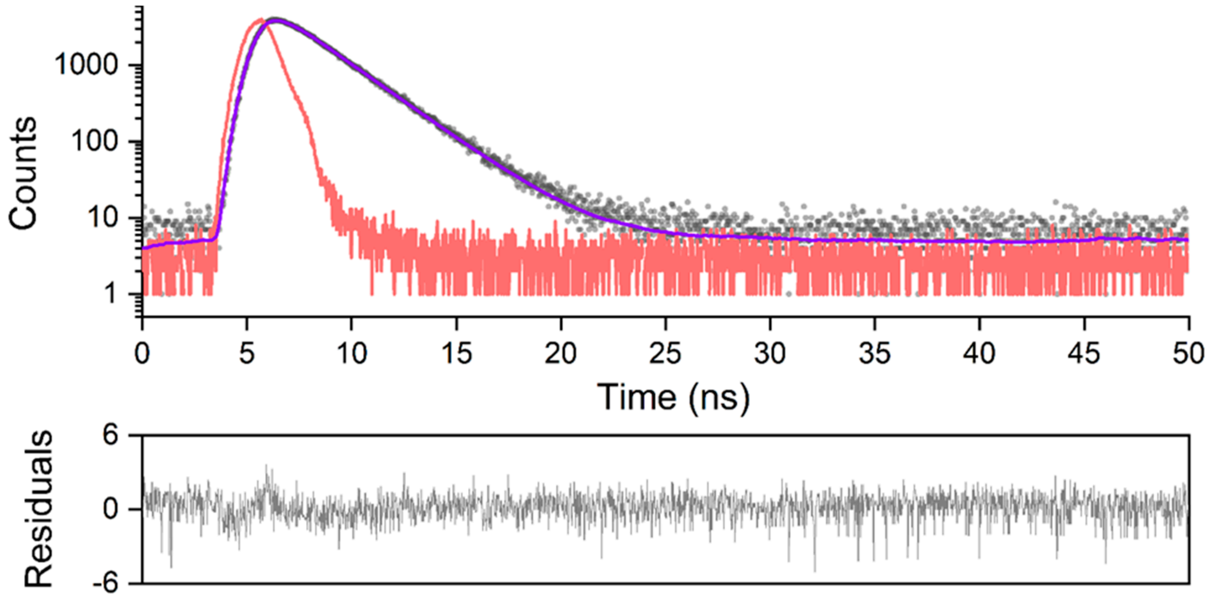

Figure S6.1. Fluorescence decay kinetics of the **HBTR-3,5-DOM** bound by **pFAST** variant. Experimental decay data (photons arrival) is represented with gray dots, the exponential fit data is shown with blue line (the monoexponential fit model was used), the instrument response function (IRF) is denoted with red curve. Residuals of fitting are shown below

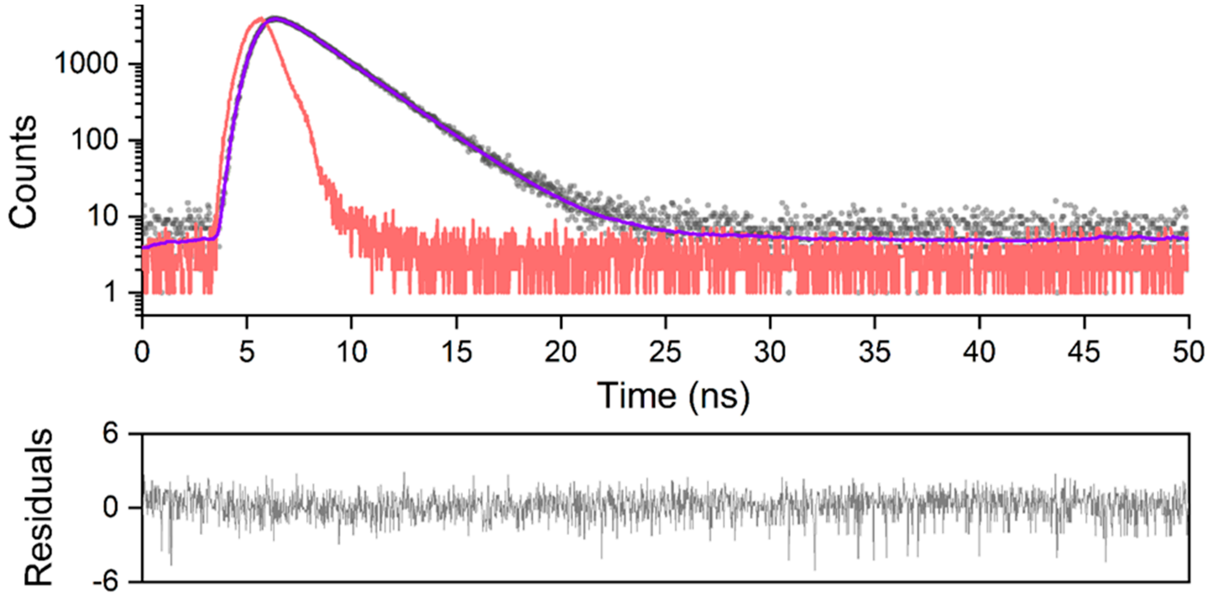

Figure S6.2. Fluorescence decay kinetics of the **HBTR-3,5-DOM** bound by **pFAST** variant. Experimental decay data (photons arrival) is represented with gray dots, the exponential fit data is shown with blue line (the biexponential fit model was used), the instrument response function (IRF) is denoted with red curve. Residuals of fitting are shown below.

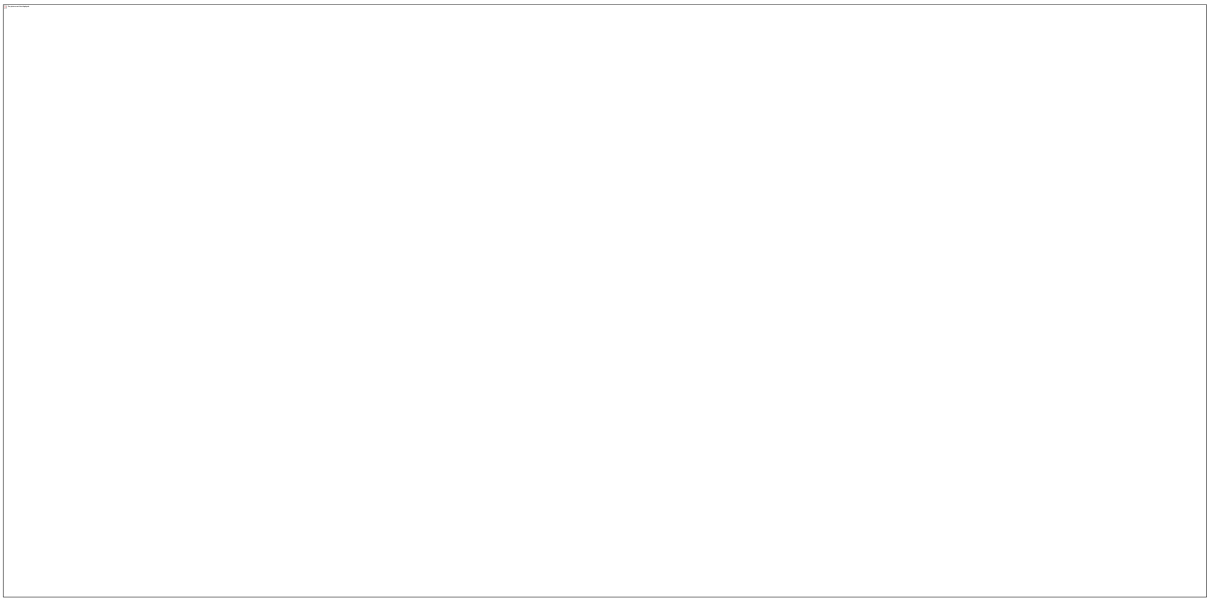

Figure S6.3. Fluorescence decay kinetics of the **HBTR-3,5-DOM** bound by **FAST-D65K** variant. Experimental decay data (photons arrival) is represented with gray dots, the exponential fit data is shown with blue line (the monoexponential fit model was used), the instrument response function (IRF) is denoted with red curve. Residuals of fitting are shown below.

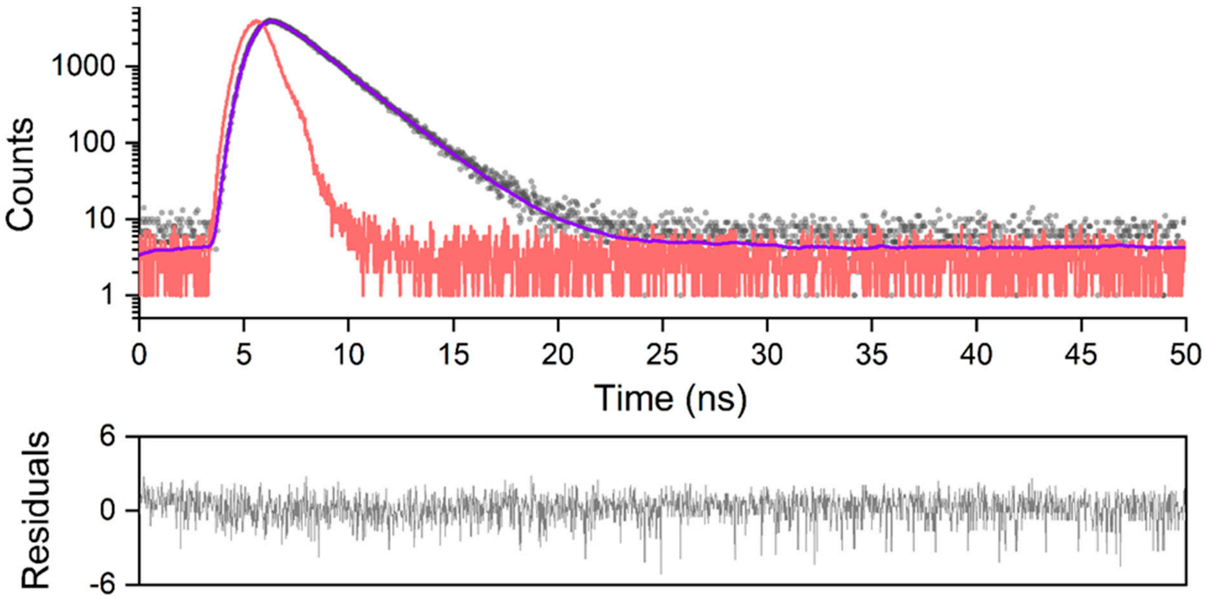

Figure S6.4. Fluorescence decay kinetics of the **HBTR-3,5-DOM** bound by **FAST-D65K** variant. Experimental decay data (photons arrival) is represented with gray dots, the exponential fit data is shown with blue line (the biexponential fit model was used), the instrument response function (IRF) is denoted with red curve. Residuals of fitting are shown below.

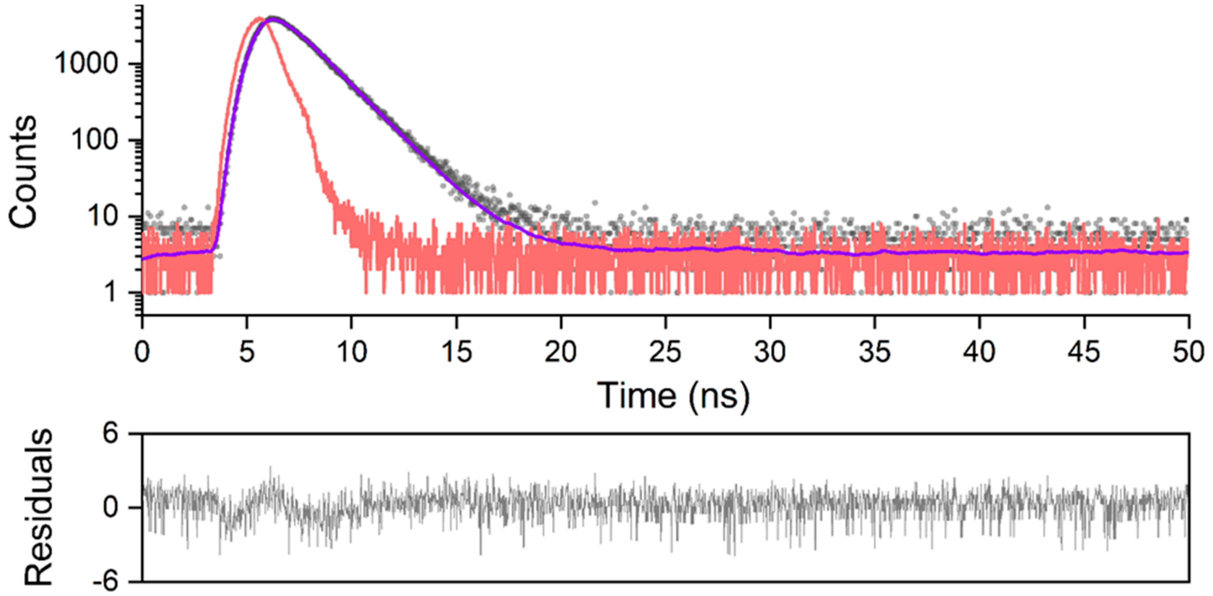

Figure S6.5. Fluorescence decay kinetics of the **HBTR-3,5-DOM** bound by **FAST-F62L** variant. Experimental decay data (photons arrival) is represented with gray dots, the exponential fit data is shown with blue line (the monoexponential fit model was used), the instrument response function (IRF) is denoted with red curve. Residuals of fitting are shown below.

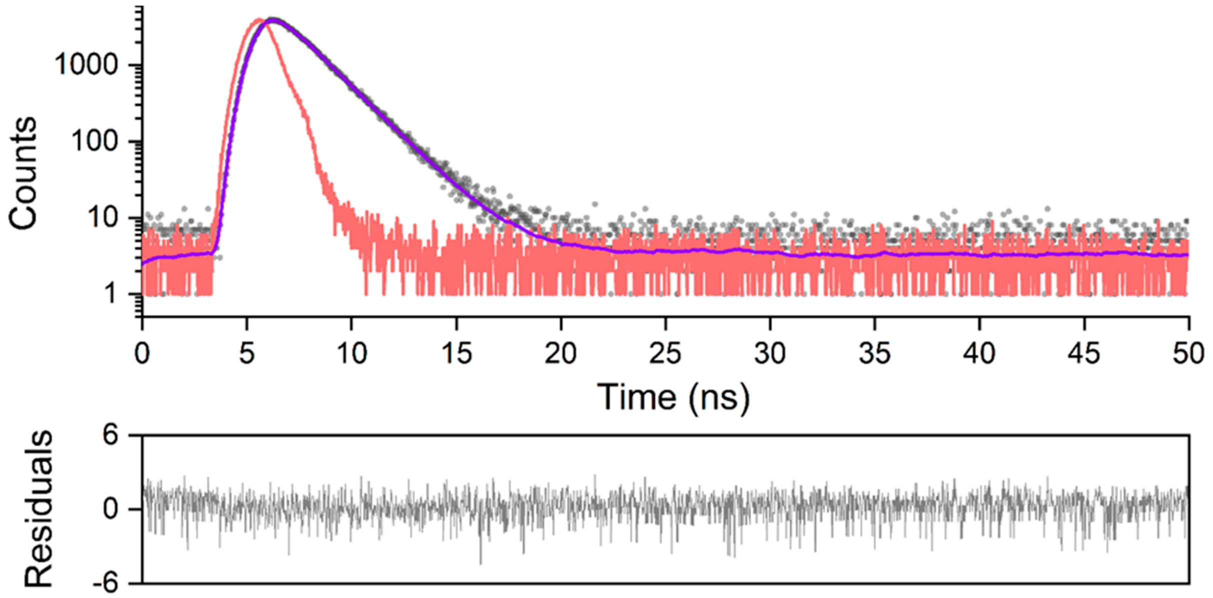

Figure S6.6. Fluorescence decay kinetics of the **HBTR-3,5-DOM** bound by **FAST-F62L** variant. Experimental decay data (photons arrival) is represented with gray dots, the exponential fit data is shown with blue line (the biexponential fit model was used), the instrument response function (IRF) is denoted with red curve. Residuals of fitting are shown below.

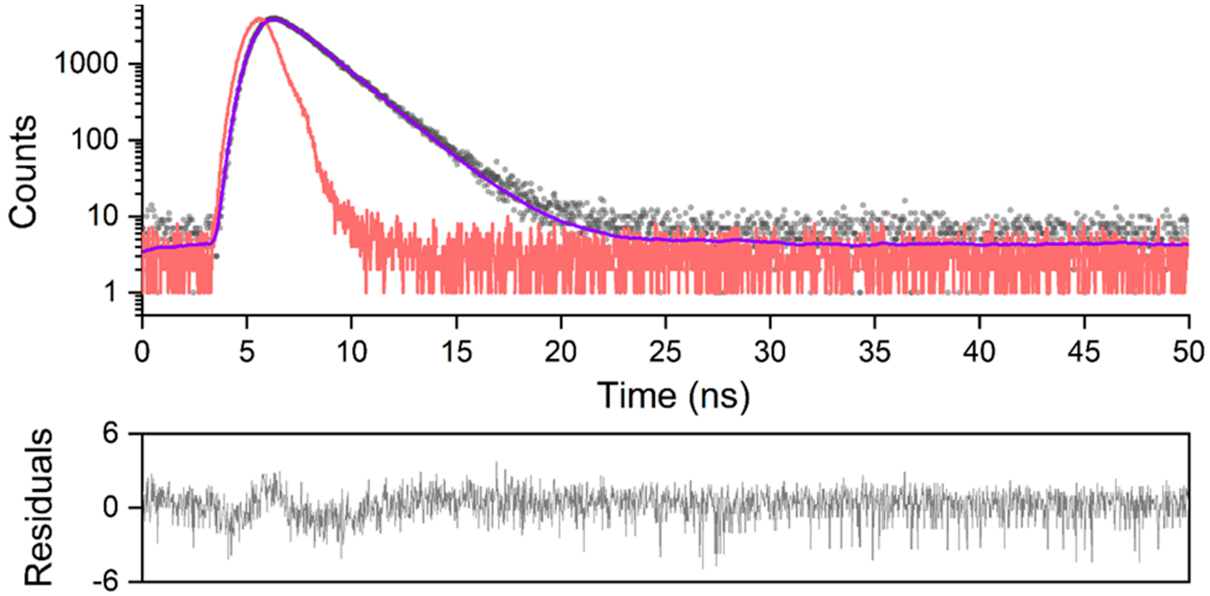

Figure S6.7. Fluorescence decay kinetics of the **HBTR-3,5-DOM** bound by **FAST-P68K** variant. Experimental decay data (photons arrival) is represented with gray dots, the exponential fit data is shown with blue line (the monoexponential fit model was used), the instrument response function (IRF) is denoted with red curve. Residuals of fitting are shown below.

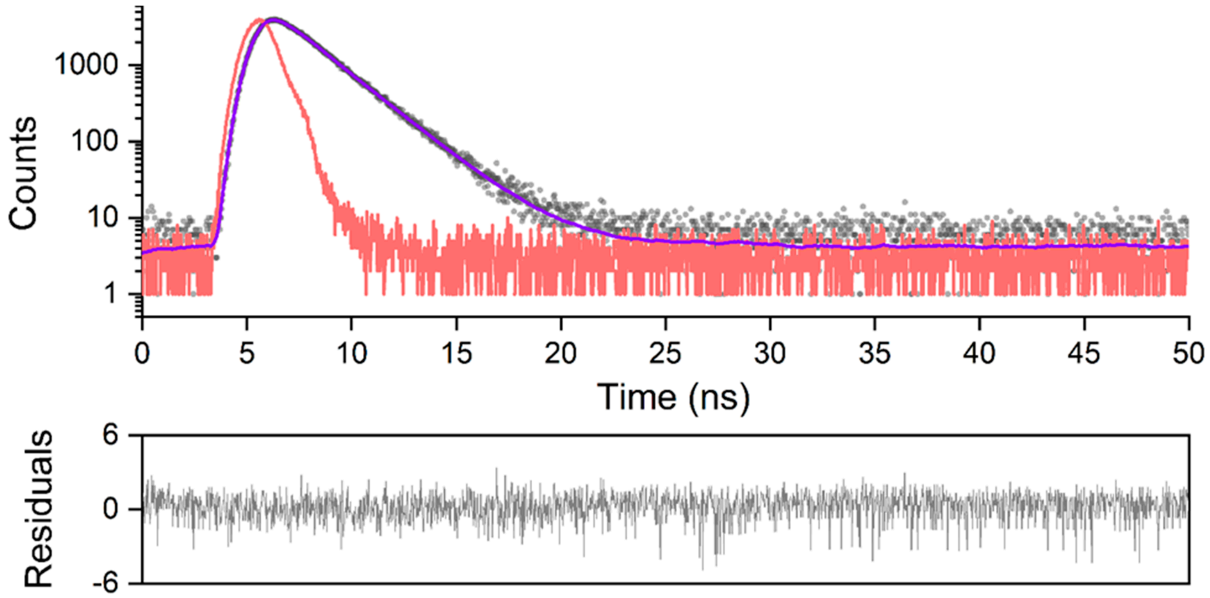

Figure S6.8. Fluorescence decay kinetics of the **HBTR-3,5-DOM** bound by **FAST-P68K** variant. Experimental decay data (photons arrival) is represented with gray dots, the exponential fit data is shown with blue line (the biexponential fit model was used), the instrument response function (IRF) is denoted with red curve. Residuals of fitting are shown below.

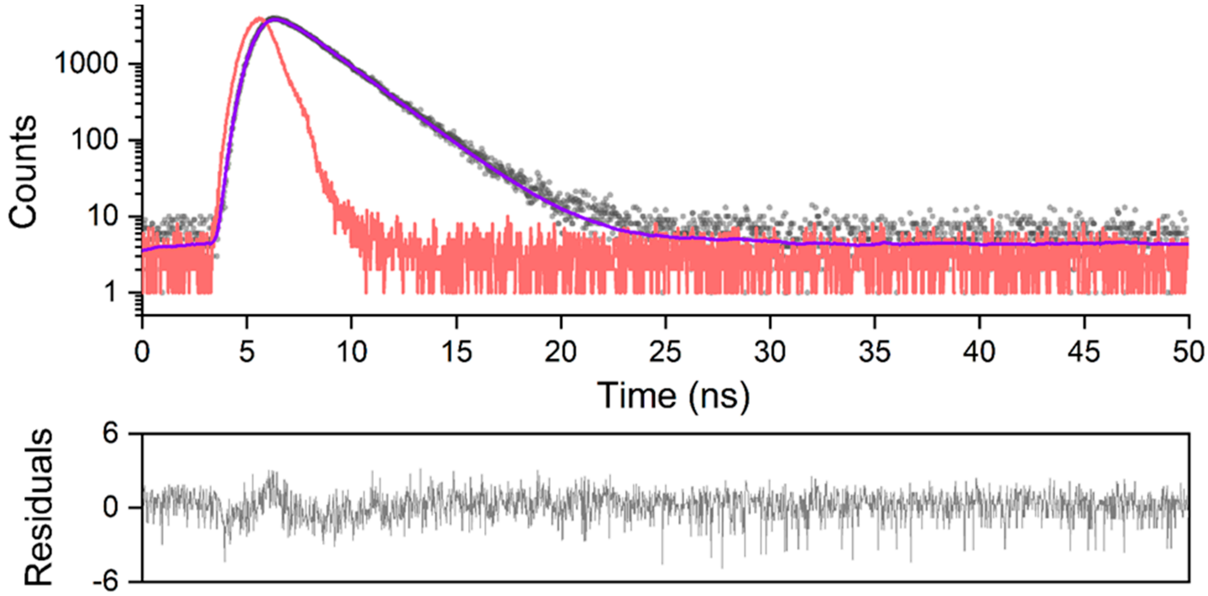

Figure S6.9. Fluorescence decay kinetics of the **HBTR-3,5-DOM** bound by **FAST-R52A** variant. Experimental decay data (photons arrival) is represented with gray dots, the exponential fit data is shown with blue line (the monoexponential fit model was used), the instrument response function (IRF) is denoted with red curve. Residuals of fitting are shown below.

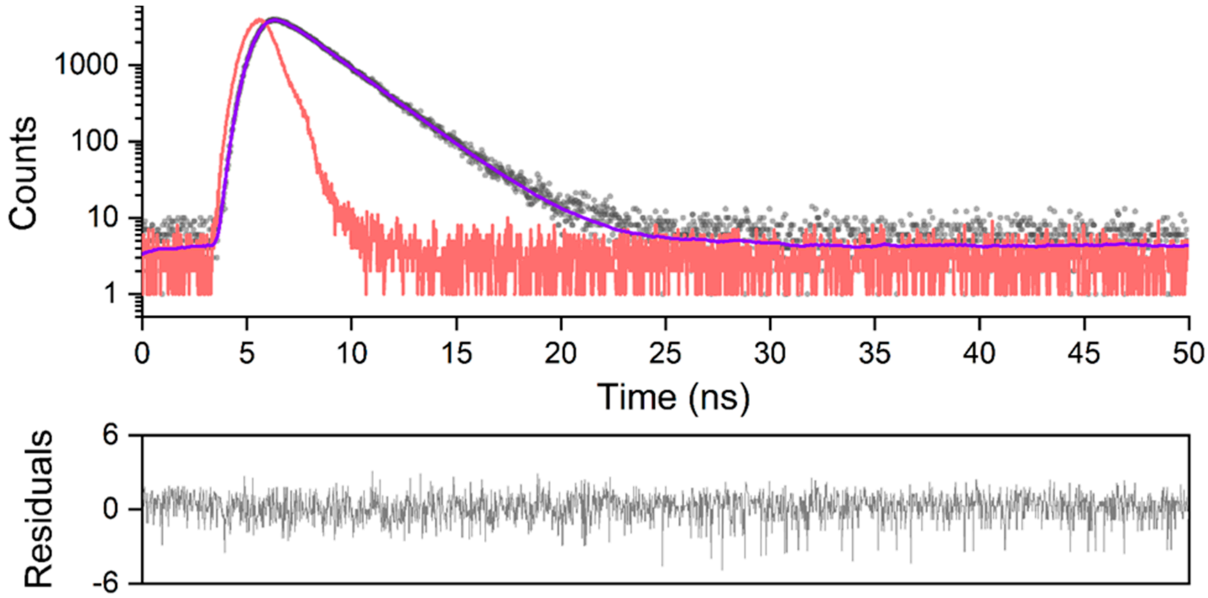

Figure S6.10. Fluorescence decay kinetics of the **HBTR-3,5-DOM** bound by **FAST-R52A** variant. Experimental decay data (photons arrival) is represented with gray dots, the exponential fit data is shown with blue line (the biexponential fit model was used), the instrument response function (IRF) is denoted with red curve. Residuals of fitting are shown below.

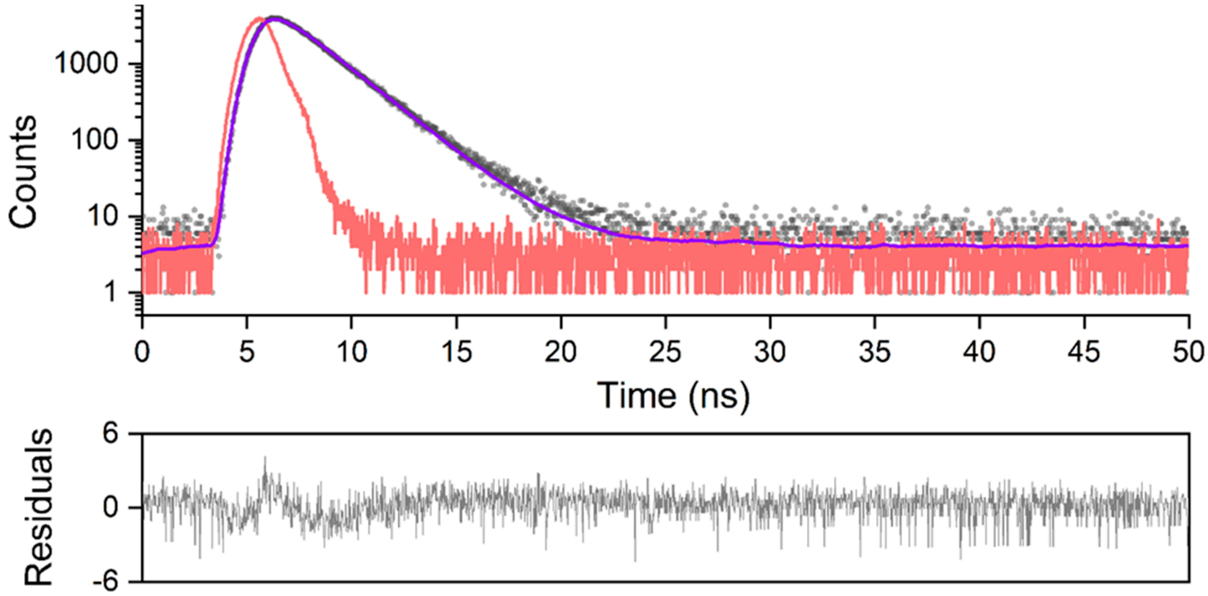

Figure S6.11. Fluorescence decay kinetics of the **HBTR-3,5-DOM** bound by **FAST-R52K** variant. Experimental decay data (photons arrival) is represented with gray dots, the exponential fit data is shown with blue line (the monoexponential fit model was used), the instrument response function (IRF) is denoted with red curve. Residuals of fitting are shown below.

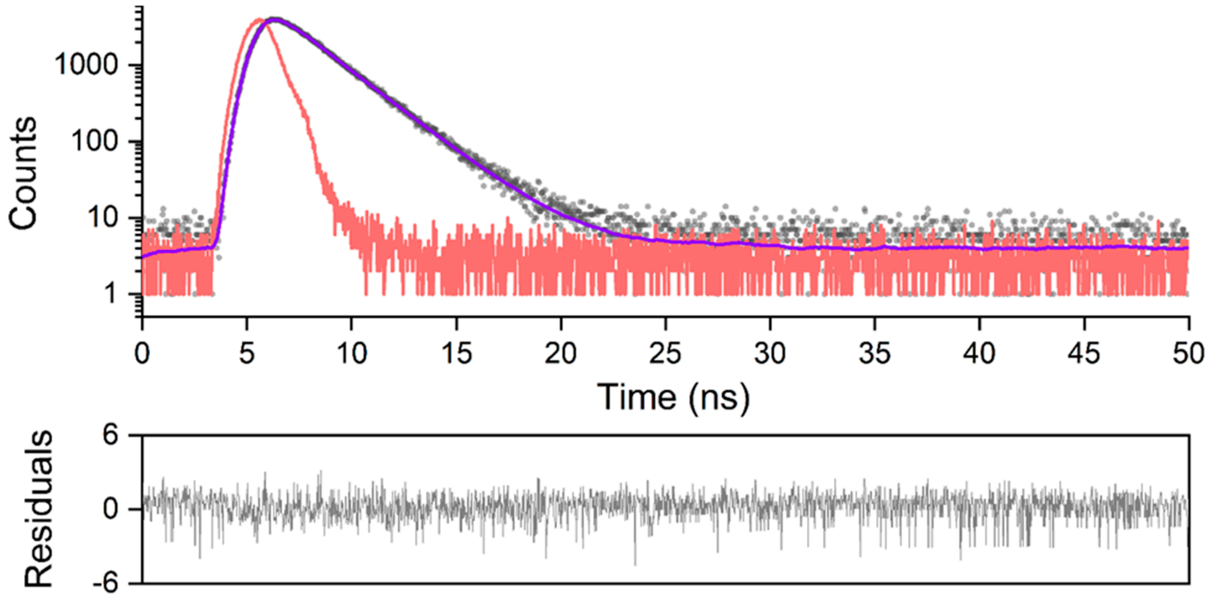

Figure S6.12. Fluorescence decay kinetics of the **HBTR-3,5-DOM** bound by **FAST-R52K** variant. Experimental decay data (photons arrival) is represented with gray dots, the exponential fit data is shown with blue line (the biexponential fit model was used), the instrument response function (IRF) is denoted with red curve. Residuals of fitting are shown below.

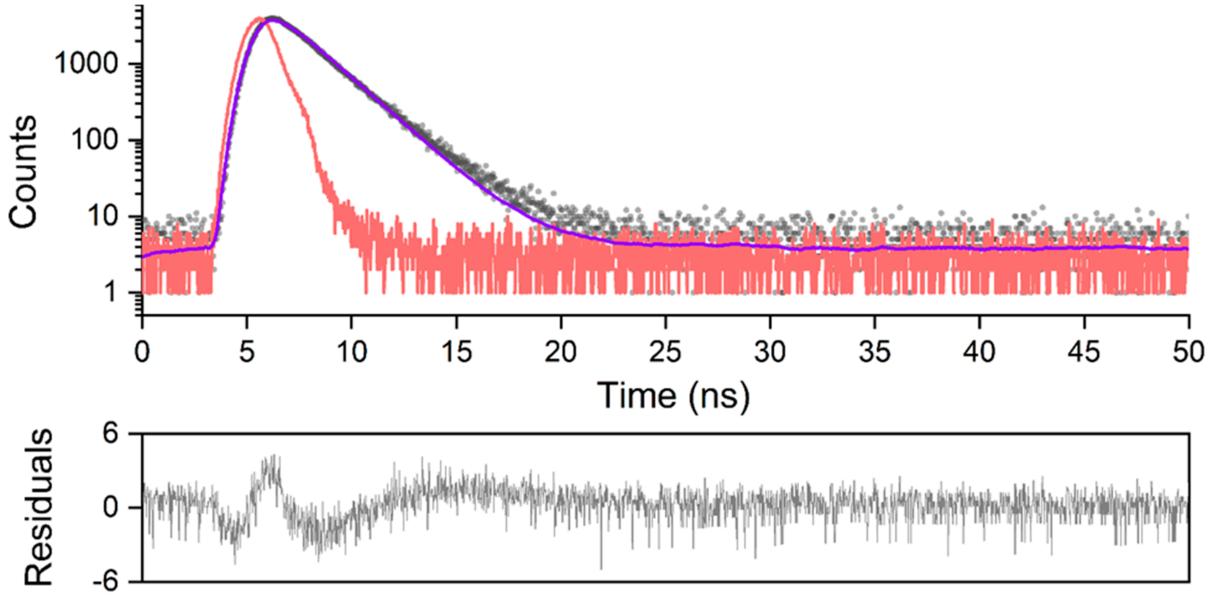

Figure S6.13. Fluorescence decay kinetics of the **HBTR-3,5-DOM** bound by **FAST-R52Y** variant. Experimental decay data (photons arrival) is represented with gray dots, the exponential fit data is shown with blue line (the monoexponential fit model was used), the instrument response function (IRF) is denoted with red curve. Residuals of fitting are shown below.

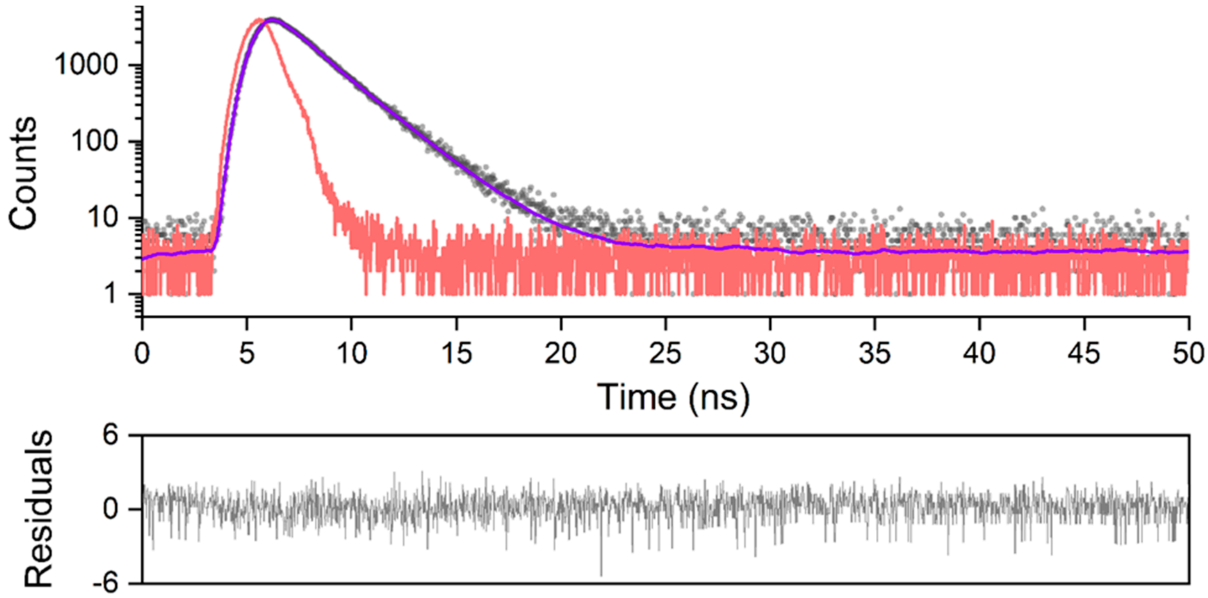

Figure S6.14. Fluorescence decay kinetics of the **HBTR-3,5-DOM** bound by **FAST-R52Y** variant. Experimental decay data (photons arrival) is represented with gray dots, the exponential fit data is shown with blue line (the biexponential fit model was used), the instrument response function (IRF) is denoted with red curve. Residuals of fitting are shown below.

7. Optical properties of the [FAST variant-HBTR-3,5-DOM] complexes

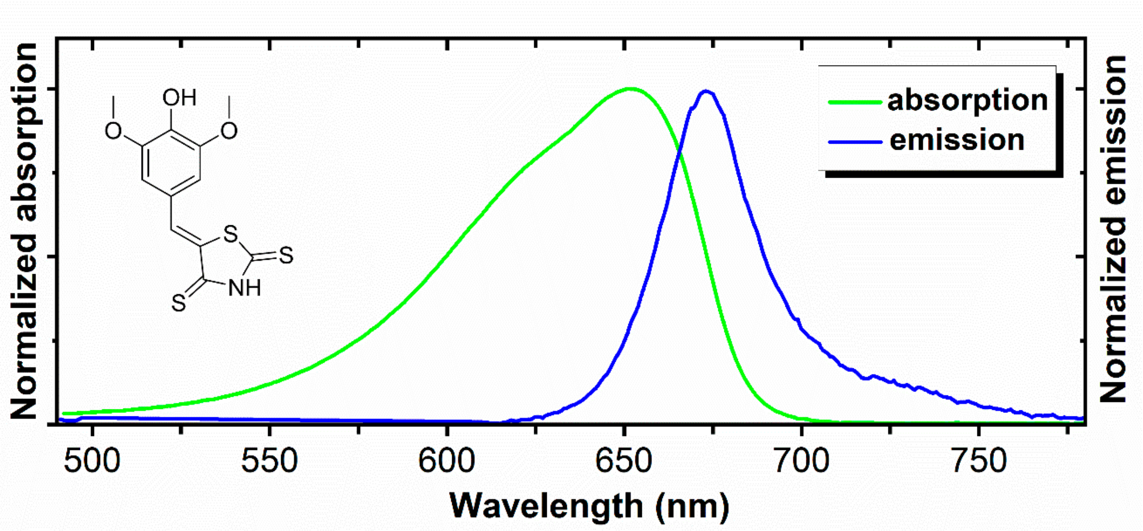

Figure S7.1. The absorption and emission spectra of **HBTR-3,5-DOM** complex with pFAST.

Figure S7.2. The absorption and emission spectra of **HBTR-3,5-DOM** complex with FAST-F62L.

8. Optical properties of the fluorogen HBTR-3,5-DOM in a free form

Table S8.1. The optical properties of the fluorogen **HBTR-3,5-DOM** in various solvents.

Compound	Structure	Solvent	ε, M^{-1}·см$^{-1}$ [a]	Abs, nm [b]
**HBTR-3,5-DOM**	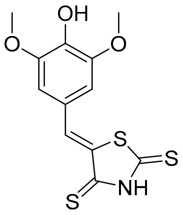	Dioxane	12500	494
		EtOAc	10000	483
		MeCN	11000	486
		EtOH	14500	471
		H_2O (pH 4)	11000	476
		H_2O (pH 11)	67000	619

[a] – absorption coefficient

[b] – absorption maximum

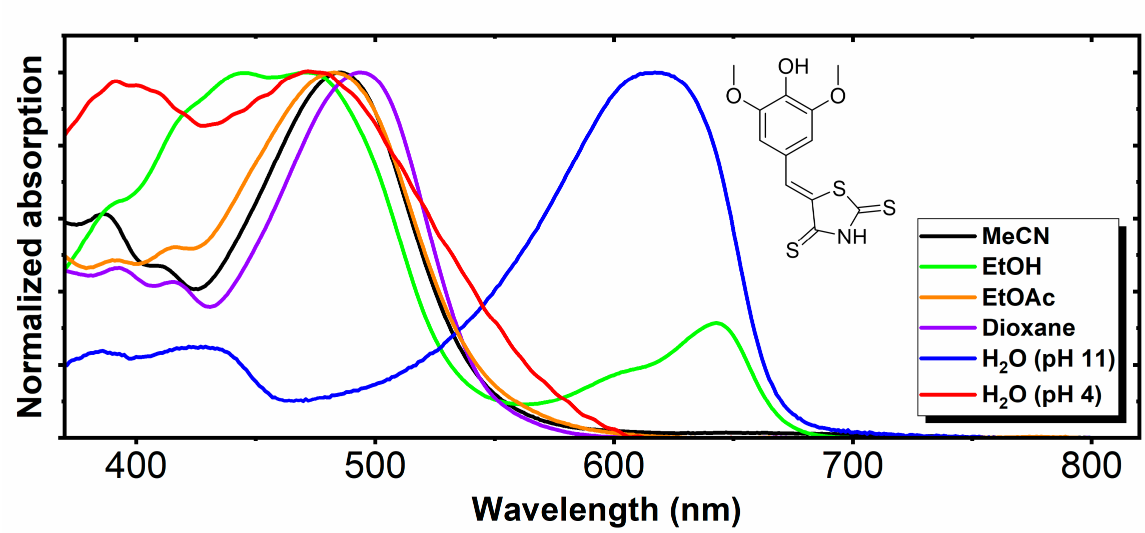

Figure S8.1. The absorption spectra of the fluorogen **HBTR-3,5-DOM** in various solvents.

9. Fluorescence anisotropy

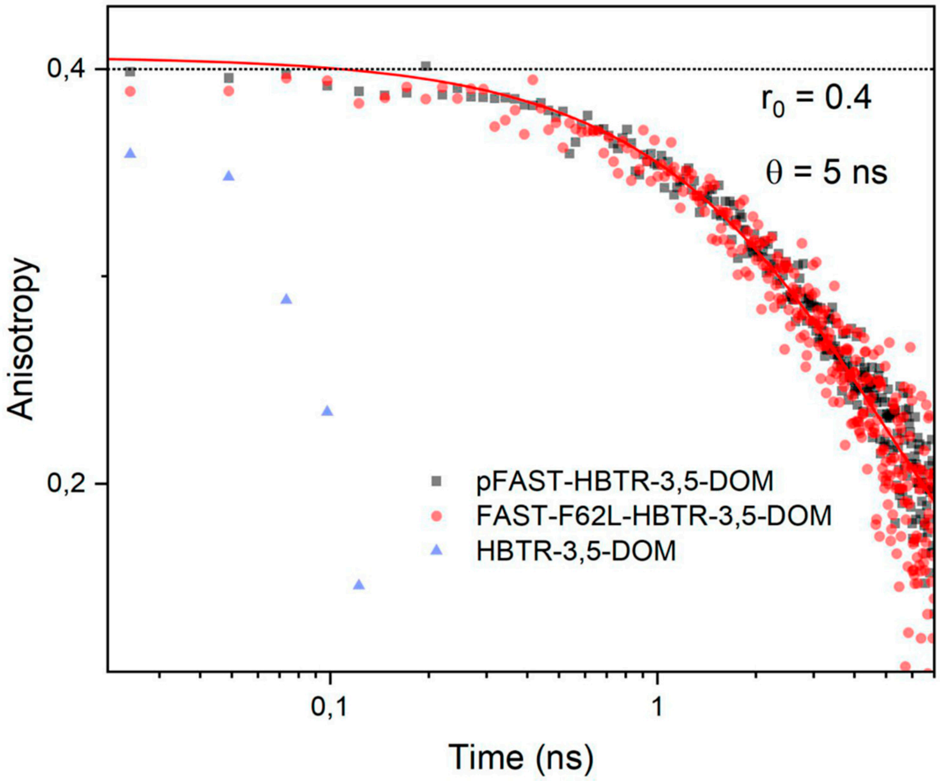

Figure S9.1. Relaxation kinetics of the fluorescence anisotropy of the HBTR-3,5-DOM dye in solution and in complexes with the pFAST and FAST-F62L proteins. Monoexponential fitting curve is shown as red line. Fluorescence was excited with 26 ps pulses at 635 nm and recorded at 675 nm. Experiments were performed at 25°C.

10. Spectrophotometric titration of fluorogens HBTR-3,5-DOM and HBTR-3-M

Table S10.1. The pK_a values of the fluorogens **HBTR-3,5-DOM** and **HBTR-3-M**.

Compound	Structure	pK_a
**HBTR-3,5-DOM**	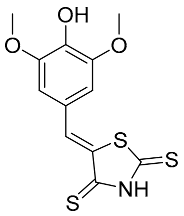	~8.0
**HBTR-3-M**	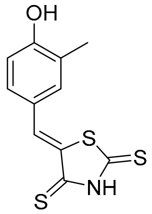	~8.0

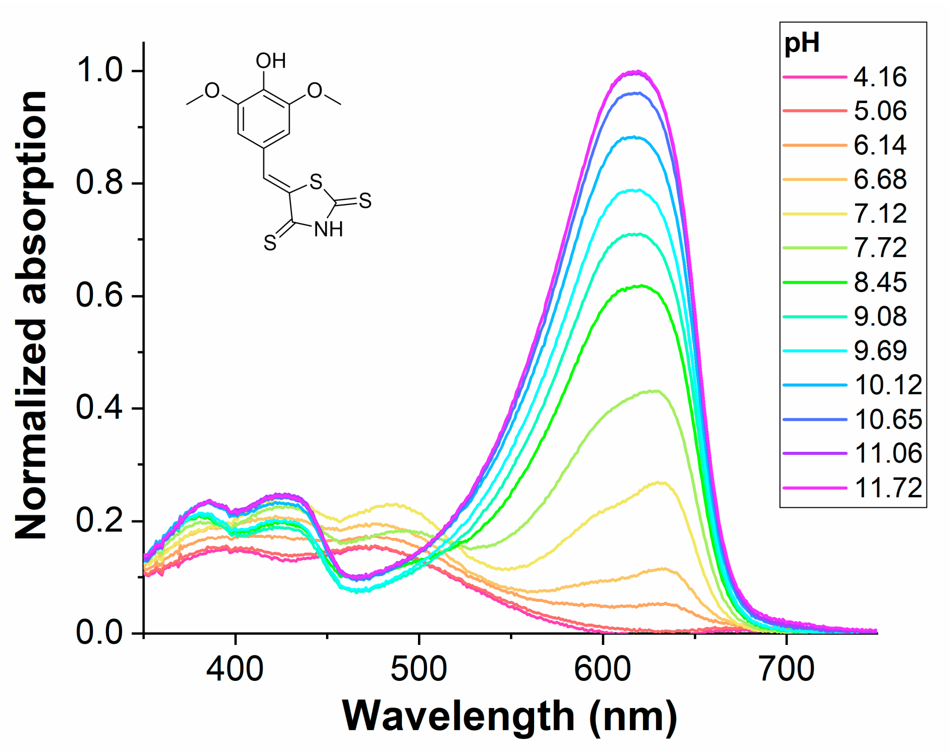

Figure S10.1. The absorption spectra of **HBTR-3,5-DOM** at various pH.

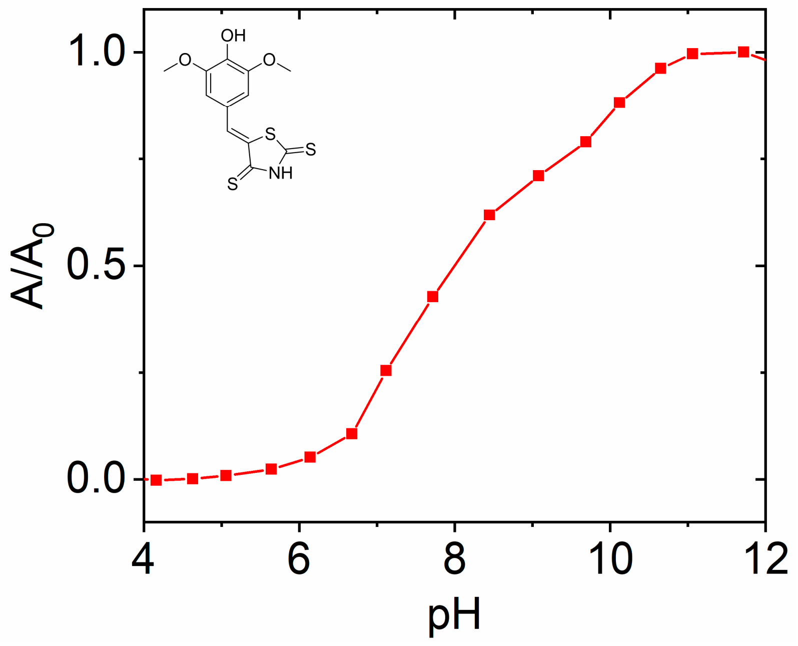

Figure S10.2. Titration curve of **HBTR-3,5-DOM** based on the absorption spectra obtained at 620 nm.

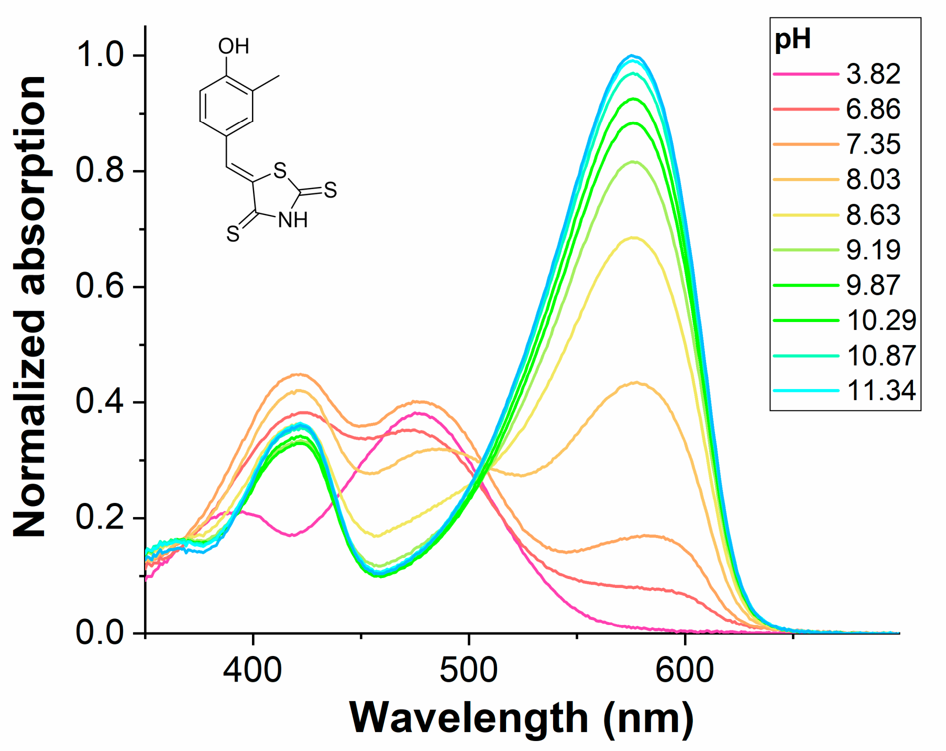

Figure S10.3. The absorption spectra of **HBTR-3-M** at various pH.

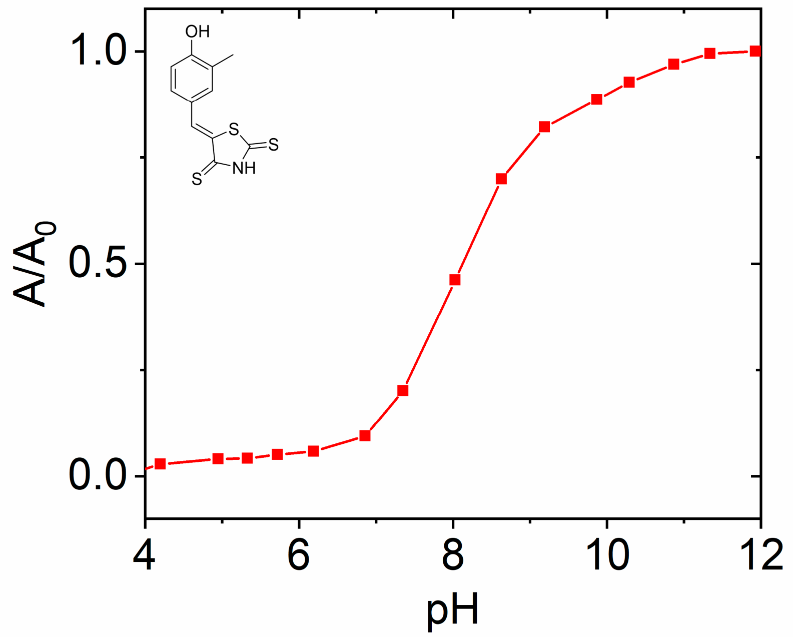

Figure S10.4. Titration curve of **HBTR-3-M** based on the absorption spectra obtained at 600 nm.

11. Cytotoxicity test

Table S11.1. The effect of **HBTR-3,5-DOM** presence in cell media on cell division.

Treatment	Initial cells number	Final cells number	Number of Initiated cell divisions	Ratio of final cells number to initial cells number	Initiated cell divisions to cells at the start ratio
**-**	257	393	106	1.53	0,41
**HBTR-3,5-DOM**, 10 μM	246	296	35	1.20	0.14
DMSO, 0.1% (v/v)	123	209	56	1.70	0.45

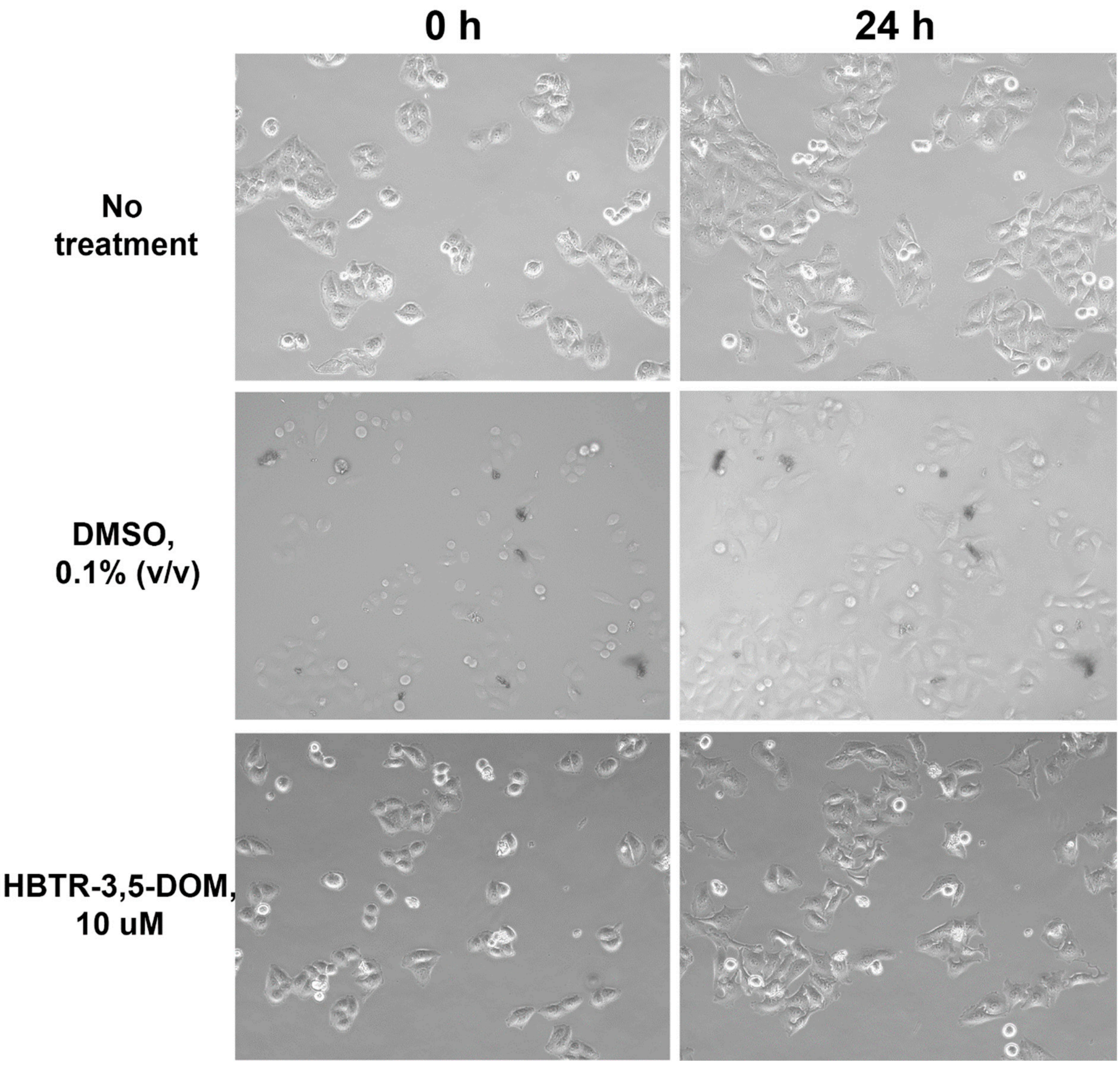

Figure S11.1. Examples of fields of view with HeLa Kyoto cells on cell culture dishes before and after treatments with **HBTR-3,5-DOM** for 24 hours.

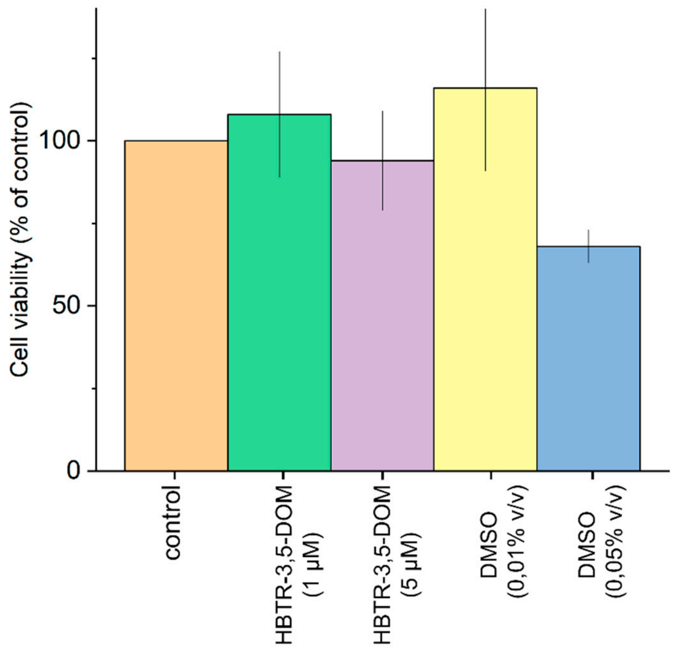

Figure S11.2. Results of MTT test. Data is shown as mean±Standard Error of the Mean.

12. FLIM

Table S12.1. Comparison of mono- and bi-exponential fits obtained *in cellulo* for complexes H2B-pFAST/H2B-F62L with **HBTR-3,5-DOM**. SD is standard deviation, in all cases the number of individual cells taken for analysis n=30.

**Fit model**	**FAST variant**	τ_1 ± SD, ns	A_1, %	τ_2 ± SD, ns	A_2, %	τ_m, ns	τ_i, ns	χ^2
**Mono-exponential**	**pFAST**	2.034±0.052	-	-	-	-	-	1.06
	**FAST-F62L**	1.474±0.044	-	-	-	-	-	1.12
**Bi-exponential**	**pFAST**	1.571±0.256	45	2.376±0.106	55	2.026±0.072	2.111±0.039	1.08
	**FAST-F62L**	1.329±0.212	47	1.566±0.073	53	1.467±0.054	1.484±0.041	1.10

Figure S12.1. H2B-FAST-F62L fusion construct in complex with **HBTR-3,5-DOM**; mono-exponential fit; τ color-coding. A screenshot from Becker&Hickl SPCImage data acquisition and analysis window is shown. On the left panel is a FLIM image of HeLa cells. A histogram on the upper right panel displays distribution of τ and color legend. The right panel represents fitting model used to fit data and fitting results. The lower data shows data on fluorescence decay. Blue dots represent experimental decay data, red line represents mono-exponential fit, green line represents instrument response function (IRF), fitting residuals shown as black graph below main data plot.

Figure S12.2. Vimentin-FAST-F62L fusion construct in complex with **HBTR-3,5-DOM**; mono-exponential fit; τ color-coding. A screenshot from Becker&Hickl SPCImage data acquisition and analysis window is shown. On the left panel is a FLIM image of HeLa cells. A histogram on the upper right panel displays distribution of τ and color legend. The right panel represents fitting model used to fit data and fitting results. The lower data shows data on fluorescence decay. Blue dots represent experimental decay data, red line represents mono-exponential fit, green line represents instrument response function (IRF), fitting residuals shown as black graph below main data plot.

Figure S12.3. IMS-FAST-F62L fusion construct in complex with **HBTR-3,5-DOM**; mono-exponential fit; τ color-coding. A screenshot from Becker&Hickl SPCImage data acquisition and analysis window is shown. On the left panel is a FLIM image of HeLa cells. A histogram on the upper right panel displays distribution of τ and color legend. The right panel represents fitting model used to fit data and fitting results. The lower data shows data on fluorescence decay. Blue dots represent experimental decay data, red line represents mono-exponential fit, green line represents instrument response function (IRF), fitting residuals shown as black graph below main data plot.

Figure S12.4. H2B-pFAST fusion construct in complex with **HBTR-3,5-DOM**; mono-exponential fit; τ color-coding. A screenshot from Becker&Hickl SPCImage data acquisition and analysis window is shown. On the left panel is a FLIM image of HeLa cells. A histogram on the upper right panel displays distribution of τ and color legend. The right panel represents fitting model used to fit data and fitting results. The lower data shows data on fluorescence decay. Blue dots represent experimental decay data, red line represents mono-exponential fit, green line represents instrument response function (IRF), fitting residuals shown as black graph below main data plot.

Figure S12.5. Vimentin-pFAST fusion construct in complex with **HBTR-3,5-DOM**; mono-exponential fit; τ color-coding. A screenshot from Becker&Hickl SPCImage data acquisition and analysis window is shown. On the left panel is a FLIM image of HeLa cells. A histogram on the upper right panel displays distribution of τ and color legend. The right panel represents fitting model used to fit data and fitting results. The lower data shows data on fluorescence decay. Blue dots represent experimental decay data, red line represents mono-exponential fit, green line represents instrument response function (IRF), fitting residuals shown as black graph below main data plot.

Figure S12.6. IMS-pFAST fusion construct in complex with **HBTR-3,5-DOM**; mono-exponential fit; τ color-coding. A screenshot from Becker&Hickl SPCImage data acquisition and analysis window is shown. On the left panel is a FLIM image of HeLa cells. A histogram on the upper right panel displays distribution of τ and color legend. The right panel represents fitting model used to fit data and fitting results. The lower data shows data on fluorescence decay. Blue dots represent experimental decay data, red line represents mono-exponential fit, green line represents instrument response function (IRF), fitting residuals shown as black graph below main data plot.

Figure S12.7. Phasor plots of **HBTR-3,5-DOM** in complexes with pFAST and FAST-F62L variants fusion construct with cellular localization signals in HeLa Kyoto cells.

Figure S12.8. H2B-pFAST and vimentin-FAST-F62L fusion constructs in complex with **HBTR-3,5-DOM**; mono-exponential fit; τ color-coding. A screenshot from Becker&Hickl SPCImage data acquisition and analysis window is shown. On the left panel is a FLIM image of HeLa cells. A histogram on the upper right panel displays distribution of τ and color legend. The right panel represents fitting model used to fit data and fitting results. The lower data shows data on fluorescence decay. Blue dots represent experimental decay data, red line represents mono-exponential fit, green line represents instrument response function (IRF), fitting residuals shown as black graph below main data plot.

Figure S12.9. H2B-FAST-F62L and vimentin-pFAST fusion constructs in complex with **HBTR-3,5-DOM**; mono-exponential fit; τ color-coding. A screenshot from Becker&Hickl SPCImage data acquisition and analysis window is shown. On the left panel is a FLIM image of HeLa cells. A histogram on the upper right panel displays distribution of τ and color legend. The right panel represents fitting model used to fit data and fitting results. The lower data shows data on fluorescence decay. Blue dots represent experimental decay data, red line represents mono-exponential fit, green line represents instrument response function (IRF), fitting residuals shown as black graph below main data plot.

13. HBTR-3,5-DOM photostability

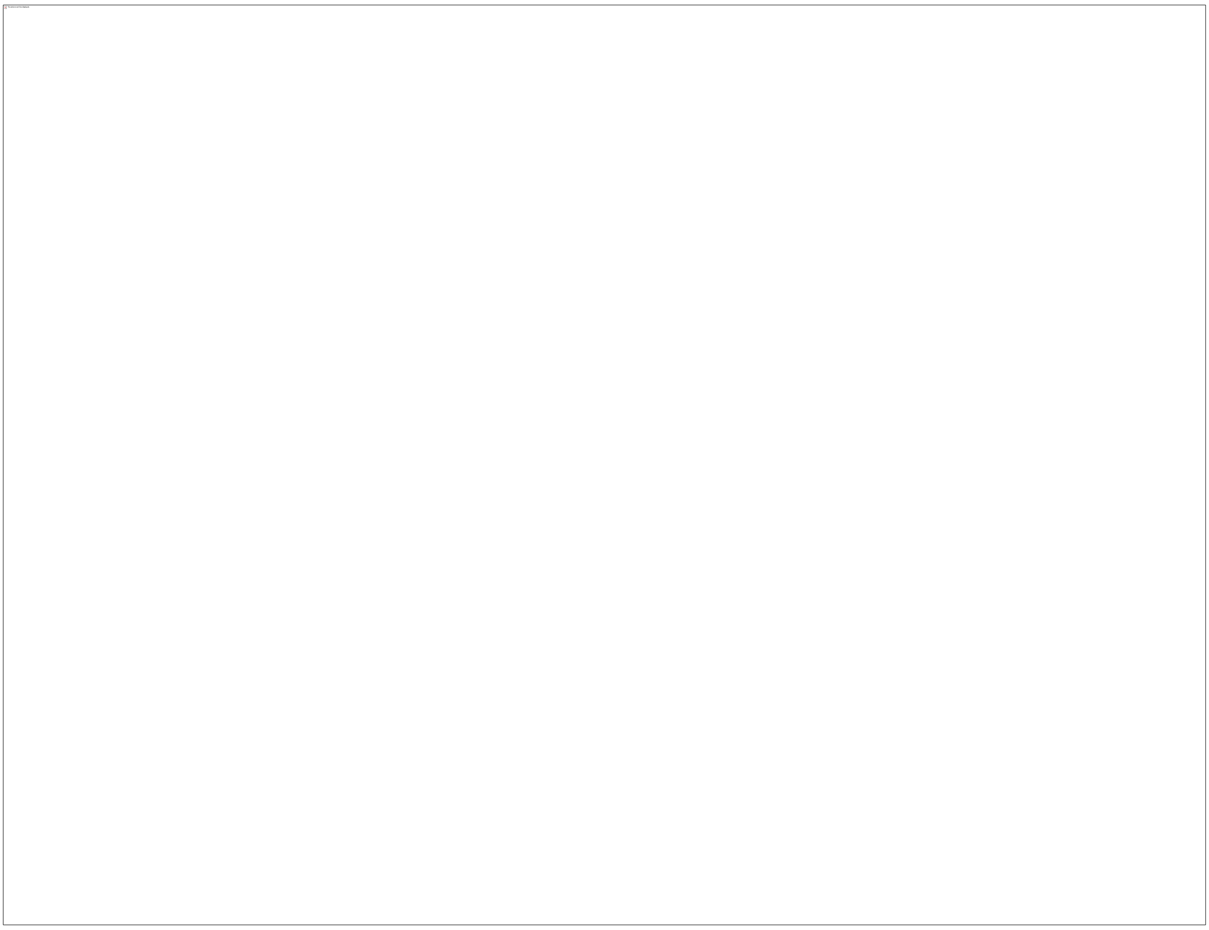

Figure S13.1. Photobleaching curves of **HBTR-3,5-DOM**, **HBR-3,5-DOM** and **N871b** in complexes with pFAST expressed as H2B fusion construct and mKate2-H2B construct in live Hela Kyoto cells. Data are shown as mean ± SD.

14. References

(1) Benaissa, H.; Ounoughi, K.; Aujard, I.; Fischer, E.; Goïame, R.; Nguyen, J.; Tebo, A. G.; Li, C.; Le Saux, T.; Bertolin, G.; Tramier, M.; Danglot, L.; Pietrancosta, N.; Morin, X.; Jullien, L.; Gautier, A. Engineering of a Fluorescent Chemogenetic Reporter with Tunable Color for Advanced Live-Cell Imaging. *Nat Commun* **2021**, *12* (1), 6989. https://doi.org/10.1038/s41467-021-27334-0.

(2) Goncharuk, M. V.; Baleeva, N. S.; Nolde, D. E.; Gavrikov, A. S.; Mishin, A. V.; Mishin, A. S.; Sosorev, A. Y.; Arseniev, A. S.; Goncharuk, S. A.; Borshchevskiy, V. I.; Efremov, R. G.; Mineev, K. S.; Baranov, M. S. Structure-Based Rational Design of an Enhanced Fluorogen-Activating Protein for Fluorogens Based on GFP Chromophore. *Commun Biol* **2022**, *5* (1), 706. https://doi.org/10.1038/s42003-022-03662-9.

15. Copies of NMR spectra

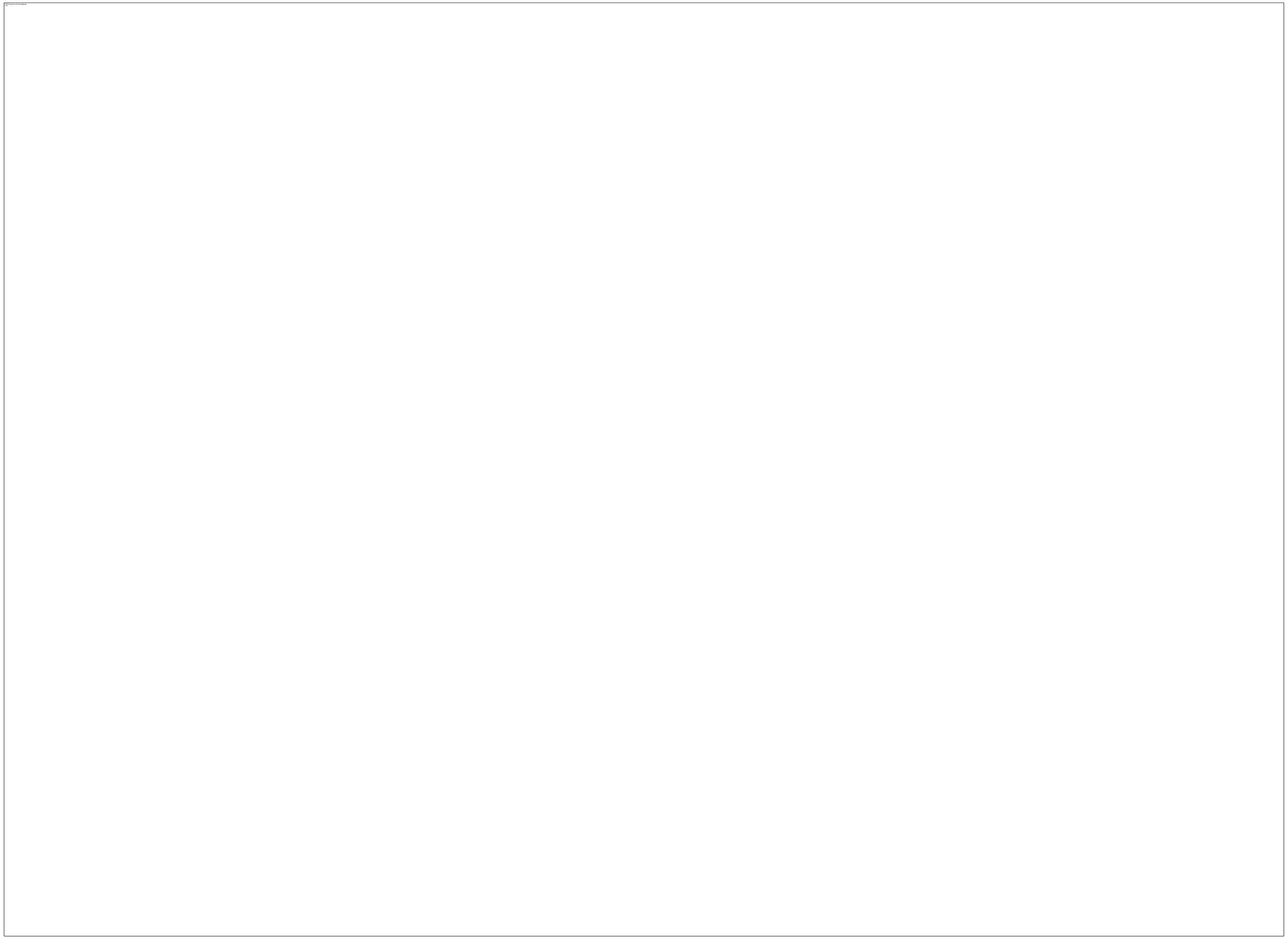

Figure S15.1. ^{1}H NMR (800 MHz, DMSO) of **HBTR-2-OM**.

The picture can't be displayed.

Figure S15.2. ^{13}C NMR (201 MHz, DMSO) of **HBTR-2-OM**.

The picture can't be displayed.

Figure S15.3. ^{1}H NMR (800 MHz, DMSO) of **HBTR-3-M**.

The picture can't be displayed.

Figure S15.4. ^{13}C NMR (75 MHz, DMSO) of **HBTR-3-M**.

The picture can't be displayed.

Figure S15.5. ^{1}H NMR (800 MHz, DMSO) of **HBTR-2,5-DOM**.

The picture can't be displayed.

Figure S15.6. ^{13}C NMR (75 MHz, DMSO) of **HBTR-2,5-DOM**.

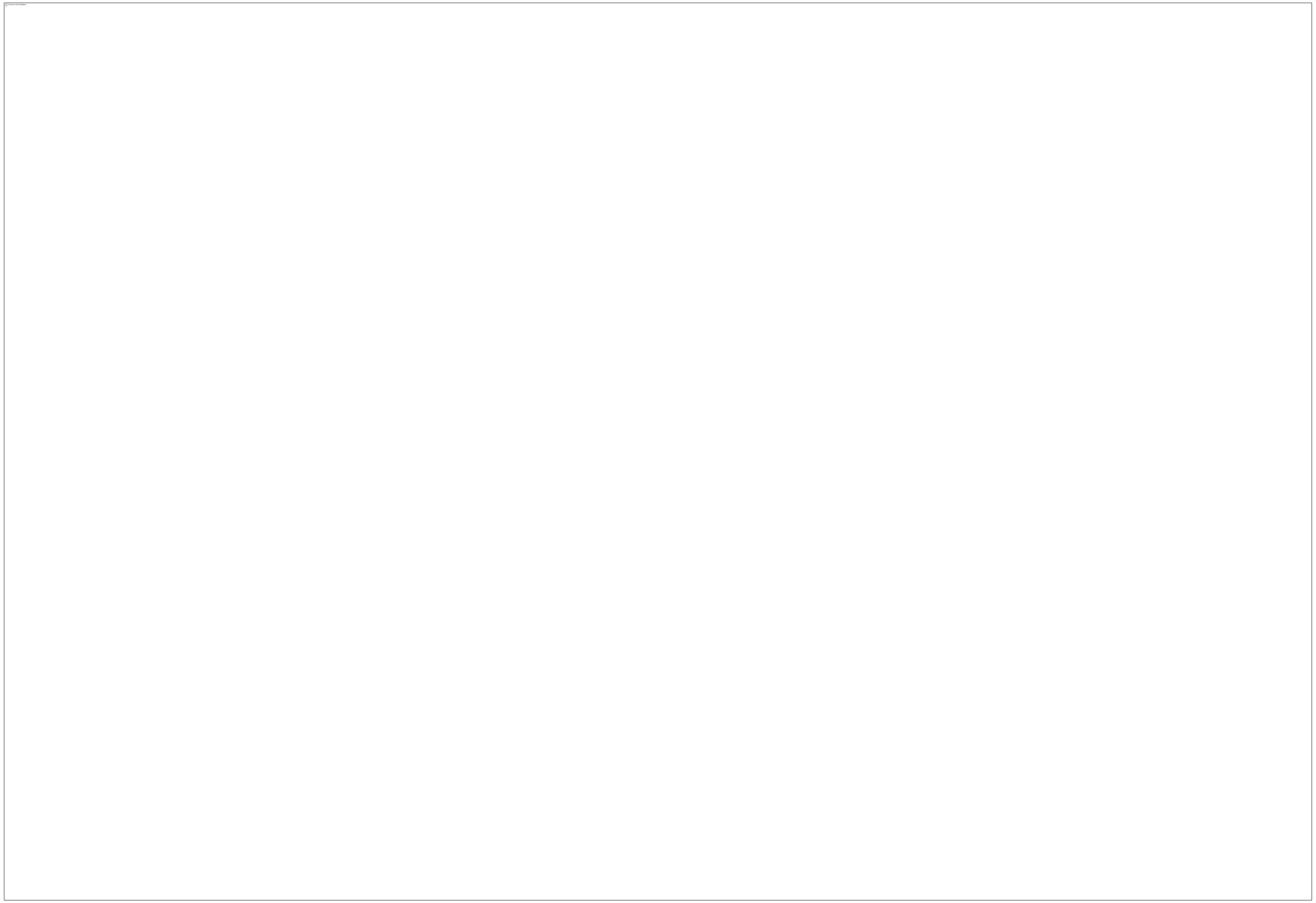

Figure S15.7. ^{1}H NMR (600 MHz, DMSO) of **HBTR-3,5-DOM** (~5mg/mL).

The picture can't be displayed.

Figure S15.8. ^{1}H NMR (600 MHz, DMSO) of **HBTR-3,5-DOM** (33 mg/mL).

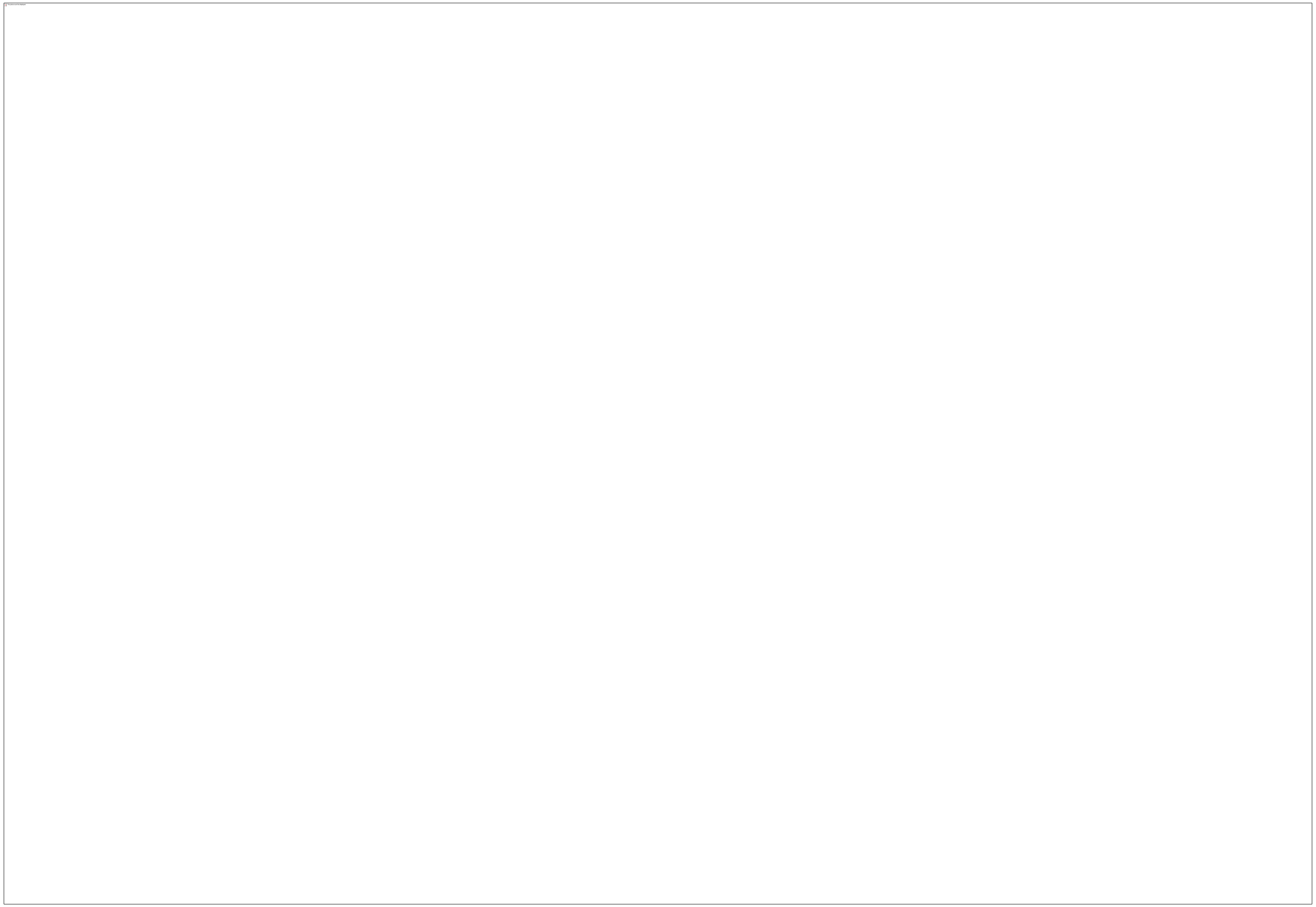

Figure S15.9. ^{1}H spectrum after dilution of a highly concentrated sample (10 times, 33 mg/mL to 3.3 mg/mL) of **HBTR-3,5-DOM**.

The picture can't be displayed.

Figure S15.10. ^{13}C NMR (151 MHz, DMSO) of **HBTR-3,5-DOM** (33 mg/mL).

The picture can't be displayed.

Figure S15.11. Key correlation in ^{1}H,^{13}C-HMBC spectrum of **HBTR-3,5-DOM** dimer.

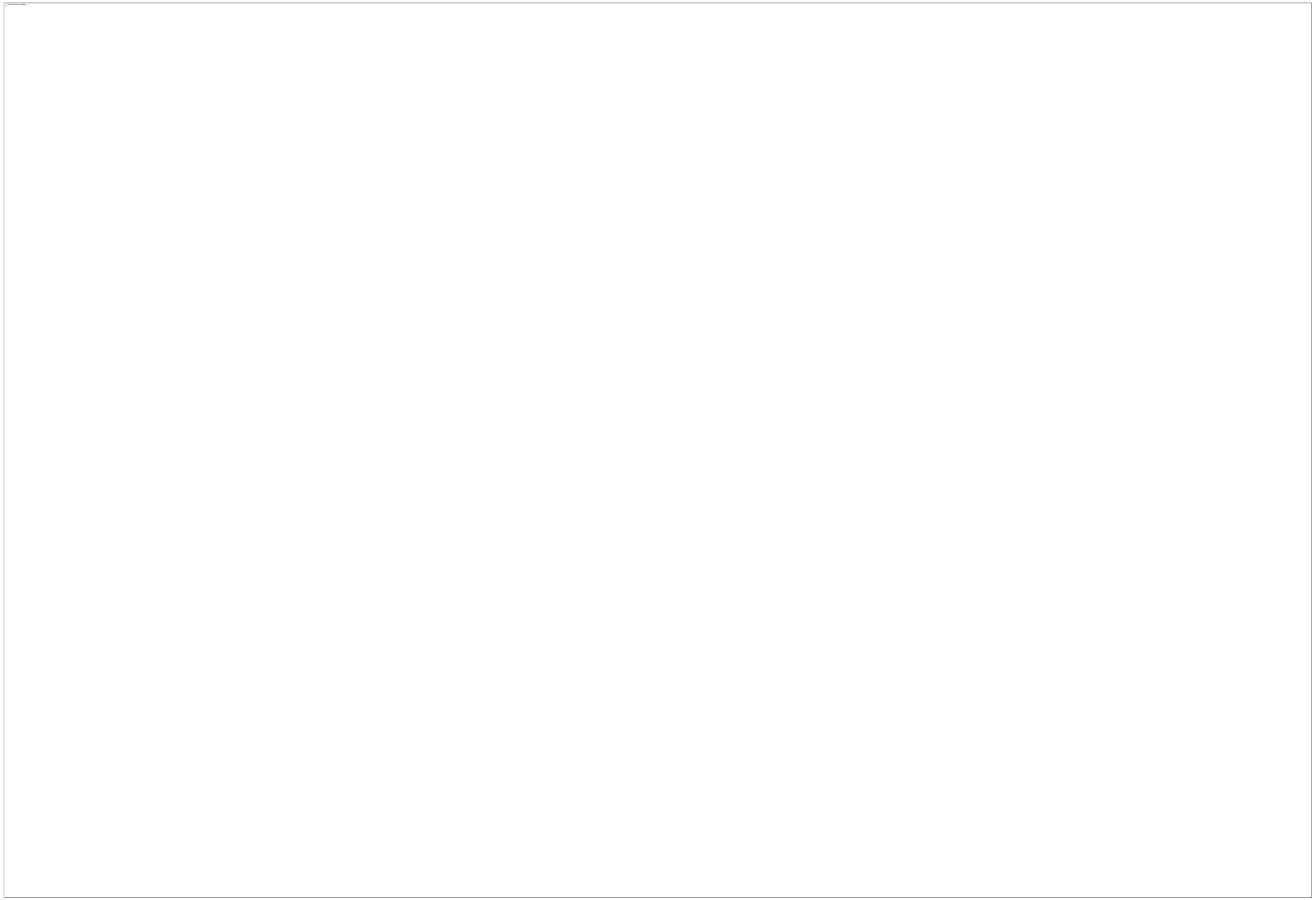

Figure S15.12. DEPT-135 NMR (151 MHz, DMSO) of **HBTR-3,5-DOM** (33 mg/mL).

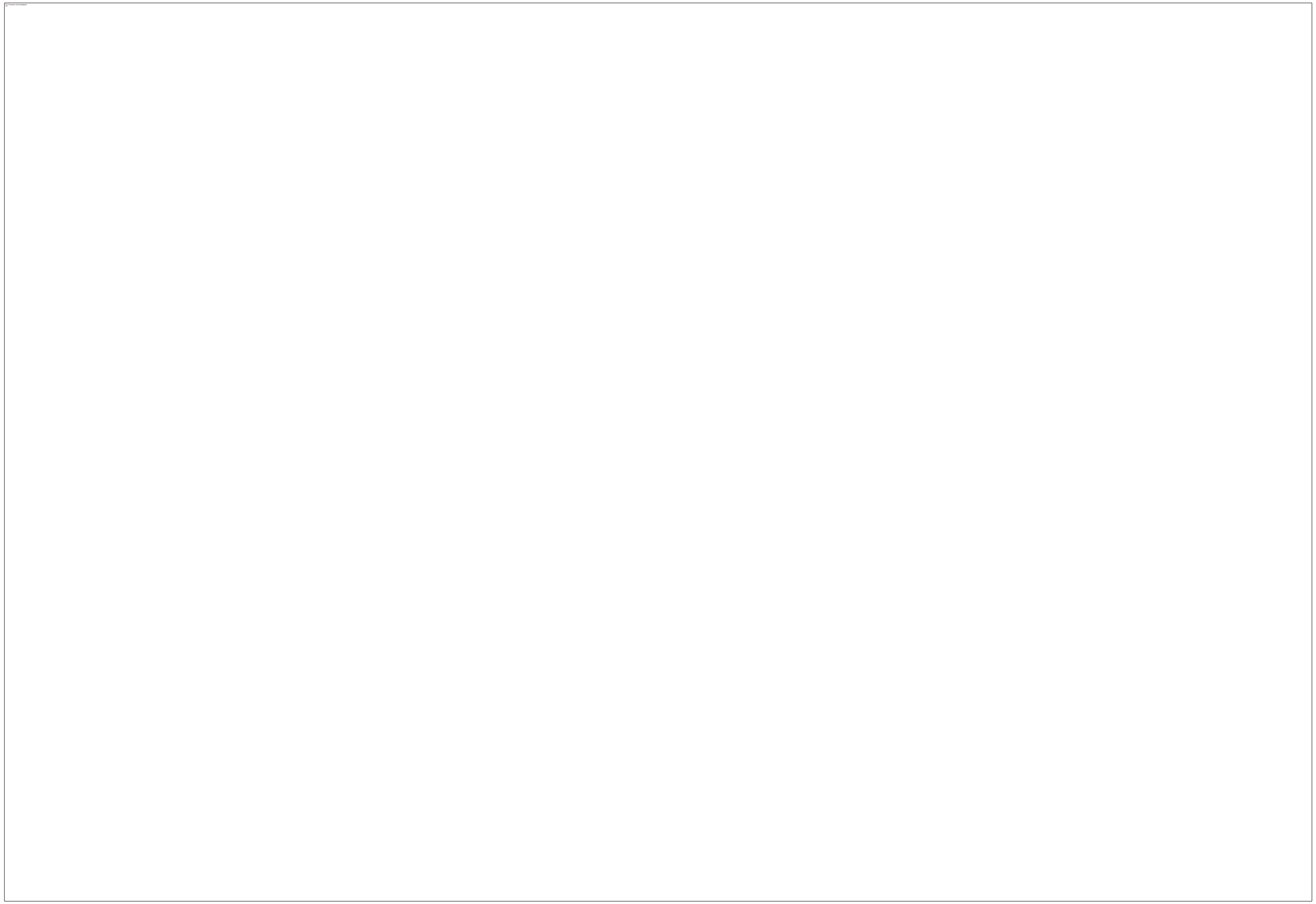

Figure S15.13. $^{1}H,^{1}H$-COSY NMR (600 MHz, DMSO) of **HBTR-3,5-DOM** (33 mg/mL).

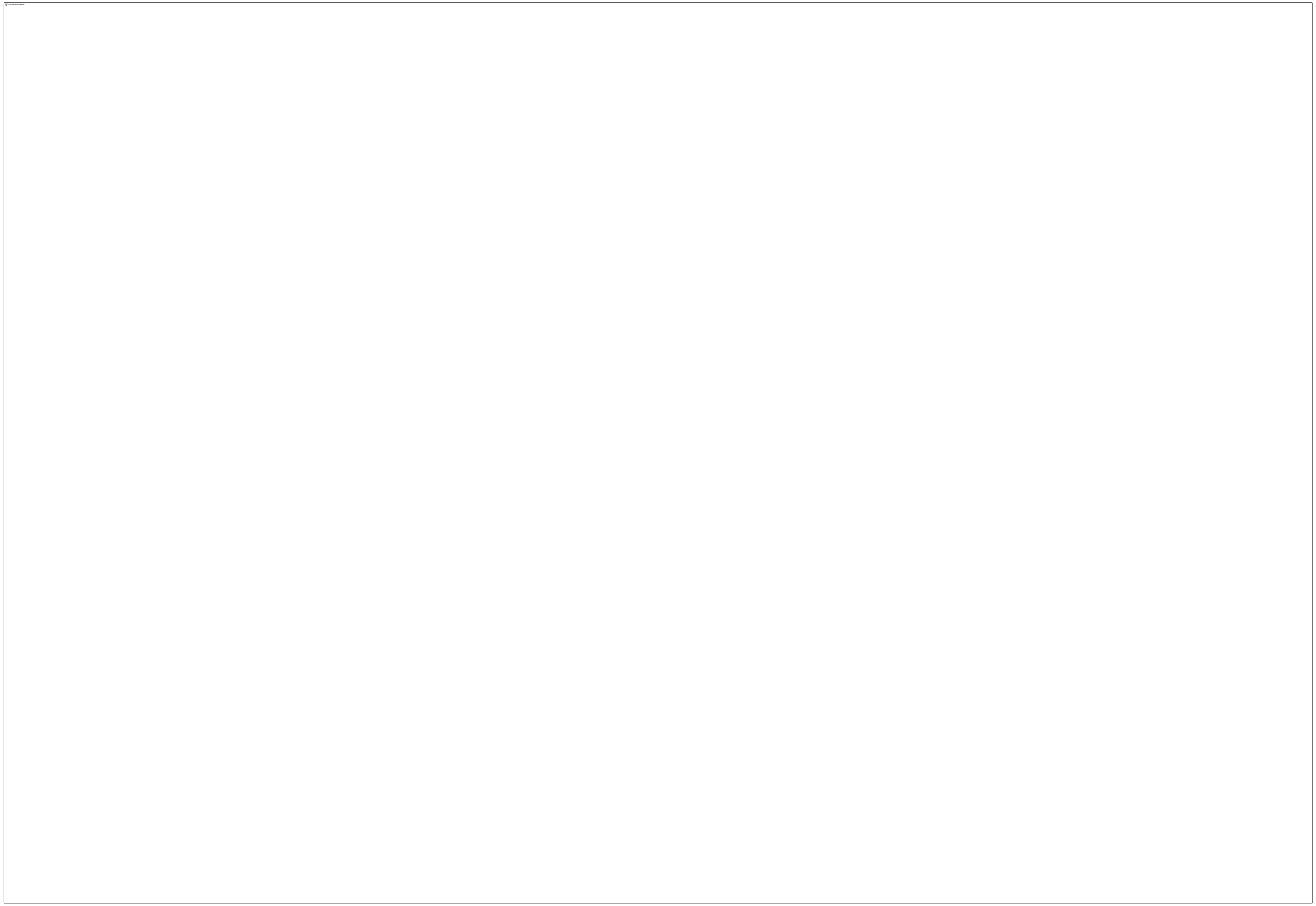
The picture can't be displayed.

Figure S15.14. $^{1}H,^{1}H$-NOESY/EXSY NMR (600 MHz, DMSO) of **HBTR-3,5-DOM** (33 mg/mL).

Figure S15.15. ^{1}H,^{13}C-HSQC NMR (F2: 600 MHz; F1: 151 MHz, DMSO) of **HBTR-3,5-DOM** (33 mg/mL).

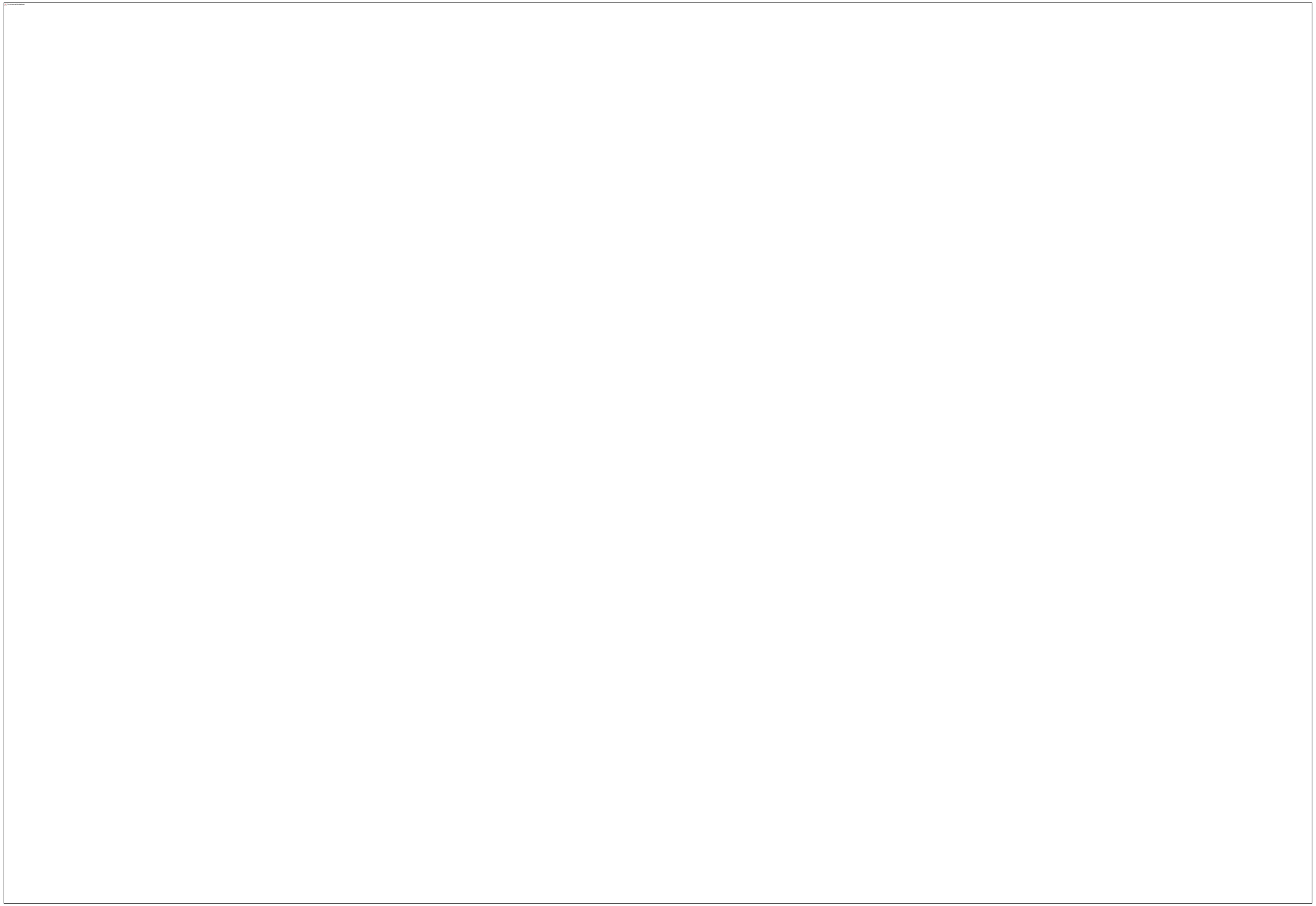

Figure S15.16. ^{1}H,^{13}C-HMBC NMR (F2: 600 MHz; F1: 151 MHz, DMSO) of **HBTR-3,5-DOM** (33 mg/mL).
